# DELAYED INPATIENT REHABILITATION AND FUNCTIONAL OUTCOMES FOR ACUTE STROKE: A RETROSPECTIVE COHORT STUDY IN AN AUSTRALIAN REGIONAL HOSPITAL

**DOI:** 10.2340/jrm.v57.42506

**Published:** 2025-08-05

**Authors:** Fan HE, Irene BLACKBERRY, Michael NJOVU, David RUTHERFORD, George MNATZAGANIAN

**Affiliations:** 1 John Richards Centre for Rural Ageing Research, La Trobe Rural Health School, La Trobe University, Victoria; 2 Melbourne School of Population and Global Health, University of Melbourne, Victoria; 3 Care Economy Research Institute, La Trobe University, Albury-Wodonga, Victoria; 4 Rehabilitation Medicine Department, Albury Wodonga Health, Wodonga, Victoria; 5 School of Clinical Medicine, University of New South Wales, Albury Campus, Albury; 6 Division of Medicine, Albury Wodonga Health, Albury, New South Wales; 7 Rural Department of Community Health, La Trobe Rural Health School, College of Science, Health and Engineering, La Trobe University, Victoria, Australia

**Keywords:** stroke, regional, Australia, delay, rehabilitation, outcomes

## Abstract

**Background:**

The impact of delayed inpatient rehabilitation on the functional outcomes of stroke patients has not been reported in regional Australia.

**Objective:**

This study examined the impact of delayed inpatient rehabilitation following acute stroke on functional outcomes (Relative Functional Gain and Functional Independence Measure efficiency) and length of stay in rehabilitation at a regional Australian hospital.

**Methods:**

Rehabilitation initiated > 24 h after a patient was deemed clinically ready was considered delayed. Associations between delayed inpatient rehabilitation and functional outcomes were investigated with mixed effects linear regression while length of stay was modelled using a negative binomial regression.

**Results:**

Of a total 487 patients, 301 (61.8%) experienced delayed inpatient rehabilitation, with a median delay of 2 days (interquartile range: 1–4 days). Multivariate regressions showed delayed inpatient rehabilitation was negatively associated with Relative Functional Gain (Beta: –0.07, 95% confidence interval [CI]: –0.11, –0.02, *p* = 0.009) and Functional Independence Measure efficiency (Beta: –0.18, 95% CI: –0.32, –0.04, *p* = 0.014), but positively associated with length of stay in rehabilitation wards (incidence rate ratio: 1.11, 95% CI: 1.02, 1.21, *p* = 0.021). Bed unavailability was the leading cause of delay.

**Conclusion:**

Delayed inpatient rehabilitation is associated with poorer functional outcomes in stroke patients. Timely access to rehabilitation is crucial for optimising recovery.

## LAY ABSTRACT

This 10-year retrospective cohort study investigated delayed inpatient rehabilitation among stroke patients at a regional hospital in Australia. It found that patients who failed to begin inpatient rehabilitation within 24 h of being deemed clinically ready had slower and poorer functional recovery. These patients also had longer stays in rehabilitation. The primary cause of delay was a shortage of available rehabilitation beds. These findings underscore the critical need for timely inpatient rehabilitation and increased bed availability to improve recovery outcomes for stroke patients in regional Australia.

Cerebrovascular diseases, including stroke, are among the leading causes of death and disability worldwide (1). Stroke rehabilitation has the potential to improve both immediate and long-term functional outcomes (2). The initial 24 to 72 h following a stroke occurrence are defined as the acute phase (3), during which clinicians prioritize stabilizing the patient’s medical condition and preventing additional cerebral damage resulting from the stroke (4). Current clinical guidelines for stroke recommend initiating rehabilitation to improve patient mobility within 24 to 48 h after stroke onset (5, 6). Early rehabilitative interventions, designed to foster neural plasticity and avert further complications after stroke, set the stage for more rigorous rehabilitation (7, 8). As the necessity for acute medical intervention reduces following the stabilization of a stroke patient’s condition, a more intensive rehabilitation programme with specific objectives should be initiated (9). These rehabilitation programmes aim to improve functional performance in preparation for the patient’s discharge from hospital (9). However, the implementation of these rehabilitation regimens may be delayed due to insufficient resources in the rehabilitation ward. While prolonged wait times for transferring stroke patients to rehabilitation services have been shown to negatively impact rehabilitation efficacy (10), it remains unclear whether delayed inpatient rehabilitation is associated with functional outcomes.

Compared with metropolitan areas, patients residing in rural and regional areas often face greater obstacles accessing timely rehabilitation services due to reduced accessibility of healthcare facilities (11) and delays in the initiation of rehabilitation following stroke. Such patients often travel long distances to major cities for rehabilitation services, which can result in physical and emotional separation from local families and support systems (12). Furthermore, the shortage of healthcare professionals in rural or regional areas, including rehabilitation therapists and specialists, limits available resources for stroke rehabilitation in these underserved areas. Reports indicate a decrease in the number of employed full-time equivalent registered clinicians per capita as remoteness increases in Australia (13). Currently, the extent of delayed inpatient rehabilitation for stroke patients in regional Australia, as well as the underlying causes of these delays, has not been reported. Given the limited representation of stroke patients from regional areas in research, there is a critical need for more studies focusing on these Australian patients.

This 10-year retrospective cohort study aimed to examine the association between delays in initiating inpatient rehabilitation and functional recovery among stroke patients during their rehabilitation stay. Additionally, the relationship between these delays and the length of the rehabilitation stay was assessed.

## METHODS

### Data sources

The Human Research Ethics Committees of Albury Wodonga Health (AWH) (HREC/73611/AWHEC-2021-256466), Australian Institute of Health and Welfare (AIHW) (EO2021/5/1311), and La Trobe University (HREC73611) approved this study using de-identified data. Patient demographic information, diagnoses, comorbidities, and admissions were extracted from routinely collected administrative data within the Electronic Medical Records system at AWH. The rehabilitation records of all included patients were provided by the Australasian Rehabilitation Outcomes Centre (AROC) through a probabilistic data linkage project conducted by AROC, which collects rehabilitation data from both public and private services in Australia and New Zealand. The linkages of acute stroke admission data from AWH and rehabilitation records from AROC are illustrated in Fig. S1.

### Sample description and study design

As a health service provider in regional Australia, AWH serves a large catchment area of 10 local governments, serving a diverse population of approximate 280,000 people, mainly of the New South Wales and Victoria border towns of Albury and Wodonga. AWH is a comprehensive regional healthcare provider offering many services including cardiovascular care, stroke treatment, maternity services, mental health and dental care (14, 15). The distance from AWH to the nearest metropolitan city, Melbourne, is approximately 320 km.

In this retrospective cohort study, patients aged ≥ 18 years who were consecutively admitted to AWH for either a first-ever or recurrent acute stroke (ischaemic, haemorrhagic, or stroke of unspecified type) between January 2010 and December 2020 were included if they had linked rehabilitation records from AROC. The AROC records included in-patient rehabilitation provided in designated rehabilitation wards and did not capture data from ambulatory or outreach rehabilitation programmes. Patients who commenced rehabilitation more than 1 year after their discharge from the index stroke admission were excluded, as were patients whose primary diagnosis was not stroke or those who died during the index admission.

The primary diagnosis of stroke in the index admission was identified using the International Statistical Classification of Diseases and Related Health Problems (10th Revision), specifically I63 (cerebral infarction) for ischaemic stroke, I60 (nontraumatic subarachnoid haemorrhage) and I61 (nontraumatic intracerebral haemorrhage) for haemorrhagic stroke, and I64 (stroke which is not specified as haemorrhage or infarction) for stroke of unspecified type. Total stroke included all 3 types of strokes in this study.

### Outcomes

The primary outcomes of this study were patient functional performance gains during the inpatient rehabilitation following acute stroke as measured by the Relative Functional Gain (RFG) and Function Independence Measure (FIM) efficiency. The FIM was assessed upon admission to, and discharge from rehabilitation. A higher FIM score indicates greater functional independence. Clinicians are trained and certified in assessing FIM to ensure consistency in FIM scoring across Australia. The RFG evaluated the patient’s functional gains after the completion of rehabilitation, relative to the maximum possible gain based on the patient’s functional level on admission to rehabilitation (16). The closer the RFG is to 1, the closer the patient is to achieving the maximum potential gain in FIM based on the functional performance level on admission. If a patient’s FIM score remains unchanged from admission to discharge, the RFG would be zero, indicating no improvement in functional independence. FIM efficiency was used to assess the daily rate of functional improvement in patients during the rehabilitation period (16). It is calculated by dividing the absolute FIM score change from rehabilitation admission to discharge by the length of stay in rehabilitation, measured in days. The RFG is calculated using the formulas published previously (15, 17). Detailed information concerning FIM and formulas for RFG calculation can be found in Appendix S1.

### Covariates

Study covariates included age on admission to rehabilitation, sex, country of birth, remoteness of residential location, indigenous status, type of stroke, stroke severity, comorbidities, delay in initiating rehabilitation, complications as documented during the rehabilitation, functional outcomes, and information on the index acute admission. Stroke severity was assessed using the National Institutes of Health Stroke Scale (NIHSS), where higher scores indicated worse neurological function (18), and by the patient’s ability to walk independently upon index admission – a validated indicator of stroke severity (19). Detailed explanations of all covariates are included in Appendix S2.

Stroke patients in the acute ward were first assessed by the supporting allied health team, which included physiotherapists, occupational therapists, speech therapists, and dietitians. During the acute phase of stroke presentation, only essential rehabilitative treatments were provided. The rehabilitation delivered in the acute ward was lower in intensity compared with the comprehensive coordinated rehabilitation offered in designated rehabilitation wards. The primary goal of these acute-phase interventions was to prevent further deterioration in patients’ condition until they transition to inpatient rehabilitation.

After admission to the rehabilitation ward, rehabilitation plans were developed following comprehensive assessments. These plans guided the rehabilitation programme, which focused on restoring motor, functional, and neurocognitive abilities affected by the stroke. This comprehensive approach was delivered by a multidisciplinary team comprising rehabilitation physicians, physiotherapists, occupational therapists, speech pathologists, social workers, and nursing staff. When needed, patients also had access to neuropsychology and clinical psychology services.

Clinical readiness for admittance to the rehabilitation ward was assessed by ensuring there were no unresolved medical or surgical conditions that required further intervention or could hinder participation in the rehabilitation ward programme. Additionally, no pending investigations could be expected to alter their rehabilitation. The evaluation of clinical readiness aimed to prevent unnecessary transfer of stroke patients to the rehabilitation ward, only to have them returned to acute care and affect their ability to engage in rehabilitation activities. The rehabilitation ward team (rehabilitation physicians, physiotherapists, occupational therapists, speech pathologists) is experienced in understanding how various medical conditions and comorbidities interact with the primary diagnosis of stroke and conducted these assessments within 24 h of receiving the electronic referral for inpatient rehabilitation through the hospital information system. These assessments were done through in-person assessments, or via telehealth or phone consultations with the acute care team and supporting allied health staff.

A delay in rehabilitation initiation was defined as starting rehabilitation more than 24 h after rehabilitation clinicians determined the patient was clinically ready. The time at which patients were assessed as clinically ready for rehabilitation was documented, along with the time they were admitted to the rehabilitation ward. The determination of delays in initiating rehabilitation was made by comparing these documented time points. The regular team meetings, involving rehabilitation physicians, physiotherapists, occupational therapists, and speech pathologists, reviewed these admitted stroke patients to reach a consensus on the reasons for the delay, and the rehabilitation plans.

Reasons for delay in starting inpatient rehabilitation included a range of predefined factors by AROC. These encompassed change in patient medical conditions, which pertained to instances where, despite being deemed ready for rehabilitation, patients experienced a sudden deterioration in health coinciding with the availability of a bed. Additionally, delays were attributed to the unavailability of rehabilitation beds, external factors that fell outside the hospital’s control, lack of equipment (such as a ceiling hoist), and disruptive behavioural problems of the patients that prevented them from engaging in the coordinated inpatient rehabilitation programme. Multiple reasons for the delay may apply to a single patient.

Patients were classified based on their motor performance levels as follows: high motor performance (baseline FIM motor scores between 51 and 91), moderate motor performance (baseline FIM motor scores between 36 and 50), low motor performance (baseline FIM motor scores between 19 and 35), and very low motor performance (baseline FIM motor scores between 13 and 18) (16).

### Statistical analysis

Patient characteristics of those who did and did not experience delayed inpatient rehabilitation were compared using a χ^2^ test or Fisher’s exact test for categorical variables and Wilcoxon rank sum test for continuous variables. Paired Wilcoxon signed-rank tests with continuity correction compared the FIM motor scores, FIM cognition scores, and total FIM scores at rehabilitation admission and discharge. Violin plots were used to visualize the distribution of RFG and FIM efficiency. Mixed-effects linear regressions modelled the RFG and FIM efficiency, with FIM motor group as the grouping variable, accounting for the variability in patients’ baseline functional performance. After assessing potential collinearity using pairwise Spearman tests, variables with a *p* ≤ 0.1 in univariate models were included in the multivariate models, which also adjusted for age, sex, stroke type, treatment in a stroke unit or Intensive Care Unit (ICU) or Coronary Care Unit (CCU) (index admission), the number of comorbidities (index admission), NIHSS (index admission), and patient’s ability to walk independently upon index admission. The variance inflation factor (VIF) was also examined to confirm the absence of multicollinearity. The coefficients of fixed effects and intercepts of random effects from the multivariate mixed-effects linear regressions were plotted to investigate the association between the investigated factors and RFG or FIM efficiency, while accounting for variability across different FIM motor groups on admission. Sensitivity analyses were conducted by excluding patients whose inpatient rehabilitation was delayed for medical reasons. Subgroup analyses for RFG and FIM efficiency were also conducted separately for ischaemic stroke, a combined group of ischaemic stroke and stroke of unspecified type, and haemorrhagic stroke.

The distribution of the length of stay (LOS) in the rehabilitation ward was investigated using a density plot to evaluate its skewness. The LOS in the rehabilitation ward was modelled using a negative binomial regression, after assessing potential collinearity using pairwise Spearman tests. Independent variables included age, sex, stroke type, treatment in a stroke unit or ICU or CCU (index admission), and number of comorbidities (index admission), NIHSS (index admission), patient’s ability to walk independently upon index admission, as well as variables with a *p* ≤ 0.1 from univariate negative binomial models. Multicollinearity in the negative binomial regression was also assessed using VIF.

Methods for assessing the fit of mixed-effects linear models and negative binomial regression are detailed in Appendix S3. Missing data were classified as “unknown” and incorporated into the statistical analyses. Statistical significance was set at *p* ≤ 0.05 (two-sided). The statistical analyses were conducted using R (R Foundation for Statistical Computing, Vienna, Austria).

## RESULTS

Of the 607 surviving stroke patients with AROC records, 23 patients were excluded as their rehabilitation episodes occurred 1 year after their index stroke admissions, leaving a final sample of 584 patients (44.3% women; median age (interquartile range [IQR] 76 [67, 83]) (Table SI). Of these, 474 (81.2%) had ischaemic stroke, 69 (11.8%) had haemorrhagic stroke, and 41 (7.0%) had stroke of unspecified types. The median number of days from stroke onset to the commencement of inpatient rehabilitation was 9 days (IQR 6, 14). Almost all patients (99%) resided in regional and rural areas. Of these, 61% came from socioeconomically disadvantaged backgrounds, with their Index of Relative Socio-economic Advantage and Disadvantage falling below the national median in Australia. A total of 210 (36.0%) were treated in a stroke unit, ICU, or CCU during their index admission. The 3 most reported comorbidities impacting rehabilitation were cardiac disease (20%), musculoskeletal conditions, including arthritis, osteoarthritis, and osteoporosis (13%), and diabetes mellitus (12%).

There were 487 patients with data on whether their inpatient rehabilitation was delayed, with 301 (61.8%) of these having experienced a delay after being evaluated as clinically ready for rehabilitation. There was a median delay of 2 days (IQR: 1 to 4 days) in starting inpatient rehabilitation. Patients who experienced delayed inpatient rehabilitation had significantly lower FIM total scores upon admission to rehabilitation (median [IQR]: 72 [50, 92]) compared with those who did not (median [IQR]: 82 [59, 96]) (*p* = 0.013 < 0.05) (Table I).

**Table I:** Patient characteristics between those with delayed rehabilitation and without

Variable	No, *n* = 186^1^	Yes, *n* = 301^1^	*p*-value^2^
Age (years)*	76 (67.3, 83.0)	77 (67.0, 83.0)	0.962
Sex*			0.161
Male	94 (50.5%)	173 (57.5%)	
Female	92 (49.5%)	128 (42.5%)	
Type of stroke*			0.053
Haemorrhagic stroke	21 (11.3%)	35 (11.6%)	
Ischaemic stroke	144 (77.4%)	250 (83.1%)	
Stroke of unspecified type	21 (11.3%)	16 (5.3%)	
Treated in a stroke unit or ICU or CCU*			0.648
No	111 (59.7%)	172 (57.1%)	
Yes	75 (40.3%)	129 (42.9%)	
Number of comorbidities*	2 (1.3, 2.0)	2 (1.0, 3.0)	0.357
Country of birth*			0.269
Australia	104 (55.9%)	147 (48.8%)	
Overseas	10 (5.4%)	23 (7.6%)	
Unknown	72 (38.7%)	131 (43.5%)	
Social economic Status*			0.215
Below median	104 (56.2%)	187 (62.3%)	
Median and above	81 (43.8%)	113 (37.7%)	
Walk independently on admission*			0.425
Yes	14 (7.5%)	20 (6.6%)	
No	101 (54.3%)	148 (49.2%)	
Unknown	71 (38.2%)	133 (44.2%)	
NIHSS group*#			0.775
Mild	–	–	
Moderate	–	–	
Severe	–	–	
Unknown	–	–	
Indigenous background*#			0.654
No	–	–	
Yes	–	–	
Modified Monash Model remoteness*			0.572
Metropolitan area/regional centre	111 (60.0%)	189 (63.0%)	
Rural area	74 (40.0%)	111 (37.0%)	
Admitted in daytime or nighttime*			0.894
Daytime	73 (39.2%)	115 (38.2%)	
Nighttime	113 (60.8%)	186 (61.8%)	
Admitted on weekday or non-weekday*			0.546
Weekday	140 (75.3%)	235 (78.1%)	
Weekend/holiday	46 (24.7%)	66 (21.9%)	
Existing comorbidity: Cardiac disease^			0.088
No	149 (80.1%)	260 (86.4%)	
Yes	37 (19.9%)	41 (13.6%)	
Existing comorbidity: Respiratory disease^			0.739
No	172 (92.5%)	282 (93.7%)	
Yes	14 (7.5%)	19 (6.3%)	
Existing comorbidity: Drug and alcohol abuse^#			0.181
No	–	–	
Yes	–	–	
Existing comorbidity: Mental health problem^			0.236
No	168 (90.3%)	282 (93.7%)	
Yes	18 (9.7%)	19 (6.3%)	
Previous history of stroke^			1.000
No	170 (91.4%)	276 (91.7%)	
Yes	16 (8.6%)	25 (8.3%)	
Existing comorbidity: Diabetes mellitus^			0.492
No	170 (91.4%)	268 (89.0%)	
Yes	16 (8.6%)	33 (11.0%)	
Existing comorbidity: Morbid obesity^			0.851
No	181 (97.3%)	295 (98.0%)	
Yes	5 (2.7%)	6 (2.0%)	
Existing comorbidity: Chronic pain^			0.749
No	181 (97.3%)	290 (96.3%)	
Yes	5 (2.7%)	11 (3.7%)	
Existing comorbidity: Cancer^			1.000
No	177 (95.2%)	287 (95.3%)	
Yes	9 (4.8%)	14 (4.7%)	
Existing comorbidity: Dementia^			0.593
No	180 (96.8%)	287 (95.3%)	
Yes	6 (3.2%)	14 (4.7%)	
Existing comorbidity: Renal failure^#			0.163
No	–	–	
Yes	–	–	
Existing comorbidity: Arthritis/osteoarthritis/osteoporosis^			0.735
No	161 (86.6%)	265 (88.0%)	
Yes	25 (13.4%)	36 (12.0%)	
Existing comorbidity: Hearing/visual impairment^			0.164
No	174 (93.5%)	291 (96.7%)	
Yes	12 (6.5%)	10 (3.3%)	
Existing comorbidity: Other^			0.077
No	150 (80.6%)	262 (87.0%)	
Yes	36 (19.4%)	39 (13.0%)	
From onset of stroke to inpatient rehabilitation (days)^	7 (5.0, 11.0)	9 (7.0, 14.0)	0.000
Employment status prior to stroke			0.000
Retired	131 (70.4%)	239 (79.4%)	
Employed	27 (14.5%)	50 (16.6%)	
Not employed	28 (15.1%)	12 (4.0%)	
Experienced complications during rehabilitation			0.168
No	155 (83.3%)	234 (77.7%)	
Yes	31 (16.7%)	67 (22.3%)	
FIM total score^	82 (59.0, 96.0)	72 (50.0, 92.0)	0.013

1 Median (IQR); *n* (%).

2 Wilcoxon rank sum test; Pearson’s χ^2^test; Fisher’s exact test.

* On stroke admission.

^ On rehabilitation admission.

# Numbers are not shown due to small number of cases, which violates the ethics requirements of reporting.

As indicated in Table II, the median and IQR of the FIM motor score on inpatient rehabilitation admission were 54 (33, 68). The median FIM cognition score on admission was 22 (IQR 15, 27), while the median FIM total score on admission was 75 (IQR 51, 93). On discharge from the rehabilitation programme, the median FIM motor score was 79 (IQR 58, 86); the median FIM cognition score was 26 (IQR 21, 31); and the median FIM total score was 105 (IQR 79, 115). All these measures demonstrated significant improvement upon completion of the rehabilitation programme. The distributions of the RFG and FIM efficiency are shown in Fig. S2.

**Table II:** Functional Independence Measure (FIM) score on inpatient rehabilitation admission and discharge from inpatient rehabilitation

Item	Inpatient rehabilitation admission^1^	Discharge^1^	*p*-value^2^
FIM motor score	54 (33, 68)	79 (58, 86)	< 0.001
FIM cognition score	22 (15, 27)	26 (21, 31)	< 0.001
FIM total score	75 (51, 93)	105 (79, 115)	< 0.001

1 Median (IQR).

2 Paired Wilcoxon signed-rank test with continuity correction.

**Fig. 1:**
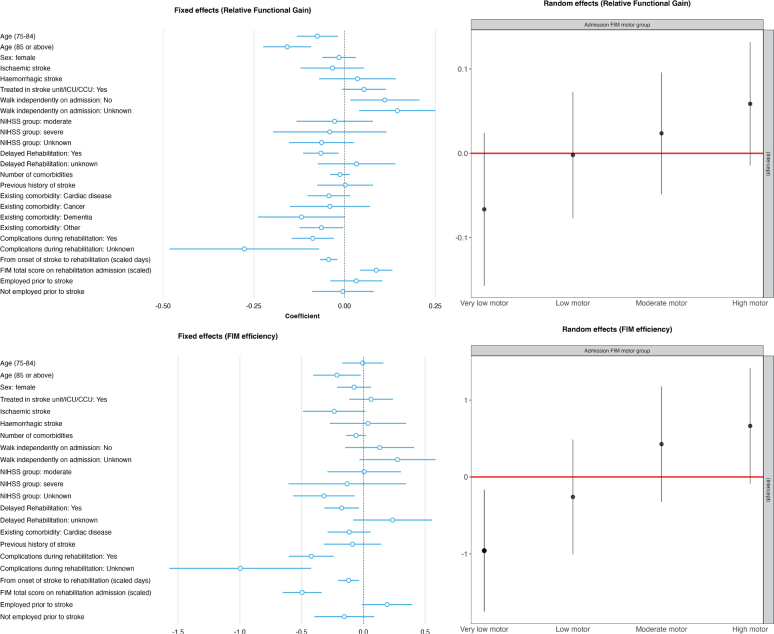
Mixed effects linear regression for Relative Functional Gain and FIM efficiency.

The multivariate regressions (Fig. 1, Table SII) that adjusted for age, sex, stroke type, ability to walk independently (index admission), NIHSS (index admission), treatment in a stroke unit, ICU or CCU, number of comorbidities, previous history of stroke, cardiac disease, cancer, dementia, other existing diseases, complications during rehabilitation, days from stroke onset to inpatient rehabilitation, FIM total score on rehabilitation admission, and employment status prior to stroke, detected a negative association between delayed inpatient rehabilitation and RFG (Beta: –0.07, 95% confidence interval [CI]: –0.11, –0.02, *p* = 0.009). Similarly, a regression (Fig. 1, Table SIII), which adjusted for a smaller set of variables, found a negative association between delayed inpatient rehabilitation and FIM efficiency (Beta: –0.18, 95% CI: –0.32, –0.04, *p* = 0.014). Older age, experiencing complications during rehabilitation, a longer time from stroke onset to inpatient rehabilitation, and a lower FIM total score on rehabilitation admission were all associated with lower RFG scores. Similarly, complications during rehabilitation and longer time from stroke onset to inpatient rehabilitation were negatively associated with FIM efficiency. Higher FIM total scores on rehabilitation admission were negatively associated with FIM efficiency. RFG and FIM efficiency varied across different FIM motor groups (Fig. 1). The diagnostic plots indicated these mixed effects linear models provided a reasonable fit to the data and that the assumptions of these models were met (Figs S3 and S4).

**Fig. 2:**
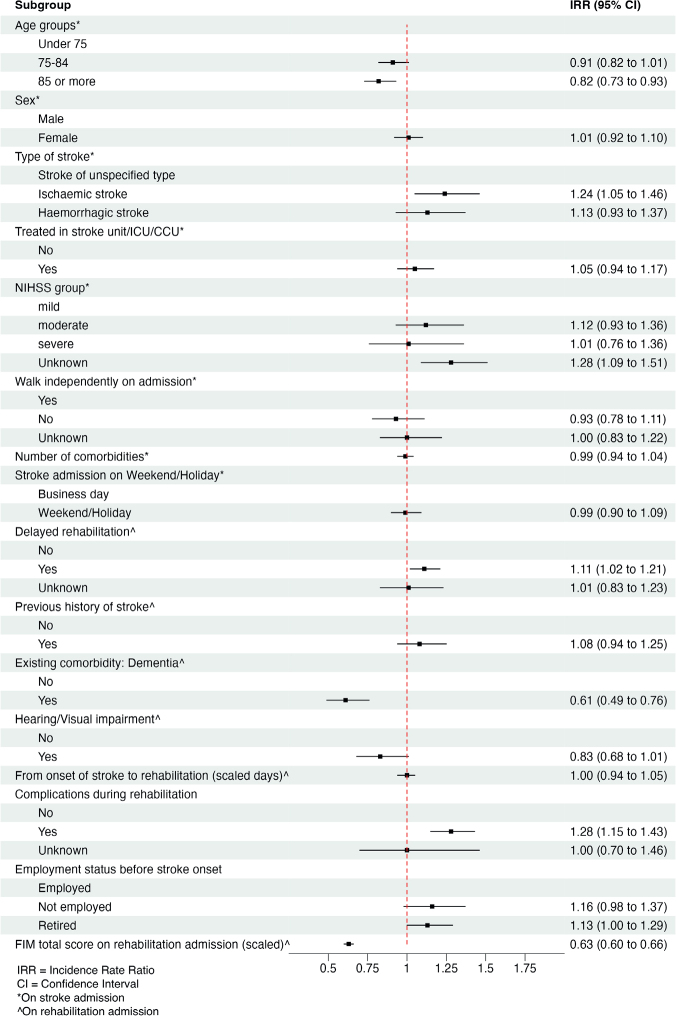
Multivariate negative binomial regression for length of stay in rehabilitation.

The LOS in the rehabilitation ward ranged from 2 to 120 days, with the median being 21 days. The density plot illustrating the LOS distribution in rehabilitation revealed a right-skewed pattern (Fig. S5). Patients who had a delayed inpatient rehabilitation were 11% more likely than those who did not have longer stays in the rehabilitation ward (incidence rate ratio [IRR]: 1.11, 95% CI: 1.02, 1.21, *p* = 0.021) (Fig. 2 and Table SIV). Similarly, ischaemic stroke and complications during admission were associated with longer stays.

Among the 301 patients who experienced delayed inpatient rehabilitation, the primary cause for 290 (96.3%) was the unavailability of beds. In contrast, only 31 (10.3%) patients had delayed inpatient rehabilitation for medical reasons.

In the sensitivity analyses (Table SV), excluding patients whose inpatient rehabilitation was delayed for medical reasons, the negative association between delayed rehabilitation and RFG remained significant (Beta: –0.07, 95% CI: –0.12, –0.01, *p* = 0.012). Similarly, the negative association between delayed rehabilitation and FIM efficiency also remained significant (Beta: –0.17, 95% CI: –0.31, –0.03, *p* = 0.020) (Table SVI). Additionally, delayed rehabilitation was associated with a longer LOS (IRR: 1.12, 95% CI: 1.03, 1.22, *p* = 0.012) (Table SVII).

In the subgroup analyses (Tables SVIII–SXIII), a significant negative association was observed between delayed inpatient rehabilitation and RFG in haemorrhagic stroke patients (Table SX). Additionally, a significant negative association was found between delayed inpatient rehabilitation and FIM efficiency in the combined group of ischaemic stroke and stroke of unspecified type patients (Table SXIII).

The outcomes of each univariate regression analysis and VIF values of variables in multivariate regressions are detailed in the Tables SXIV–SXVI, and Appendices S4–S6. The associations between the actual or stratified number of days delayed in starting rehabilitation and RFG, FIM efficiency, and LOS in rehabilitation ward were also analysed. Detailed results were provided in Tables SXVII-SXX.

## DISCUSSION

This study found that stroke patients who experienced delayed inpatient rehabilitation at a major Australian regional health service provider were more likely to have poorer outcomes, including reduced RFG and FIM efficiency. Additionally, these patients tended to stay longer in the rehabilitation ward. These findings indicate the potential negative implications for stroke patients’ clinical outcomes when inpatient rehabilitation is delayed.

Stroke patients undergo a recovery process involving neuronal regeneration, neuroplasticity, and adaptation (20). Rehabilitation plays a crucial role in facilitating all these 3 mechanisms (20, 21). There is a “plastic window”, a period of increased neuroplasticity observed after a stroke, when the brain’s ability to adapt and respond to injury is significantly heightened, making it more receptive to rehabilitation efforts (8, 22). While the exact timing and duration of the plastic window have not been definitively established in stroke patients (8), it is well understood that the timing of rehabilitation is crucial. This study has shown that delaying the start of inpatient rehabilitation can potentially affect the outcomes for stroke patients. Stroke survivors often face multifaceted impairments, such as impaired upper extremity function, loss of mobility, and aphasia (8). These challenges require a goal-oriented approach with comprehensive interventions, including mobility and functional exercises, swallowing and communication therapy, visual rehabilitation, and nutritional support, as recommended by clinical guidelines (23). Patients who experienced delays in inpatient rehabilitation may possibly miss the critical period for receiving comprehensive care in designated rehabilitation wards, hindering their rehabilitation progress and potentially impacting their overall ability to regain lost functions and prolonging their inpatient rehabilitation stay. This could have led to the reduced RFG reported in this study. Furthermore, as the length of stay increased, which served as the denominator in calculating FIM efficiency, it further decreased FIM efficiency.

Patients who experience delays in rehabilitation can undergo spontaneous recovery while waiting for a rehabilitation bed. However, there is a lack of evidence to suggest that this spontaneous improvement is significant among stroke patients with complex, multifaceted impairments, which typically require targeted therapies to address specific domains of impairment (23). While we cannot rule out the possibility that patients in this study could have undergone spontaneous recovery during the waiting period, leading to a plateau effect during subsequent inpatient rehabilitation and resulting in reduced RFG and FIM efficiency, it remains speculative. Additionally, it is noteworthy that patients who experienced delays had lower FIM scores on admission to inpatient rehabilitation. This could be attributed to the rehabilitation ward prioritizing patients with higher FIM scores (lower dependence and need for assistance) when rehabilitative resources were limited, as these patients typically required less intensive rehabilitation. However, the adjustment for the initial FIM scores in regression analyses ensured that the observed reductions in RFG and FIM efficiency were not confounded by this factor.

This study reports that unavailability of rehabilitation beds was the primary factor causing delays in rehabilitation. This contrasts with factors outside the hospital system, equipment, or patient-related concerns such as behavioural or medical problems, implying that delays in starting rehabilitation can be prevented by optimizing hospital services. Inequality in access to rehabilitation services and healthcare professionals exists between metropolitan regions and regional or rural areas in Australia, with people in non-metropolitan areas having reduced access (24). It has been reported that hospitals in regional and rural areas have significantly fewer rehabilitation beds compared with those in metropolitan areas (25). The availability of rehabilitation beds can be restricted by financial constraints, space limitations, and the number of available healthcare professionals (26). Among these factors, the recruitment of rehabilitation therapists in rural areas presents a complex challenge, influenced by factors such as a lack of established career pathways and a shortage of professional support (27). As a result, hospitals in regional and rural areas often have fewer specialized disciplines per rehabilitation unit compared with metropolitan hospitals (25). To improve bed availability in these areas, a comprehensive strategy that includes financial, policy, and career support for healthcare professionals is necessary to strengthen inpatient rehabilitation services in regional Australia.

Delayed inpatient rehabilitation can impose an additional burden on an already strained healthcare system in regional Australia, leading to a cascading effect and increased costs. As published previously (28), delayed inpatient rehabilitation is associated with lengthier stays during rehabilitation following stroke. The cascade effect occurs when patients experiencing delayed inpatient rehabilitation occupy rehabilitation units for extended periods, reducing bed availability. This, in turn, hinders the timely admission of subsequent patients, subjecting them to delayed inpatient rehabilitation as well (29). Moreover, due to the reduced capacity in rehabilitation units, patients who are clinically stable and ready to commence inpatient rehabilitation end up occupying beds in acute care, which increases hospital costs per patient. Occupying an acute care bed when acute care services are no longer medically necessary is considered a delayed discharge (30). Delayed discharge from acute care wards then delays admissions for new patients (30), particularly those living in regional areas that already have a strained healthcare system. Thus, it is important to prevent this cascade effect by reducing the number of patients who experience delayed inpatient rehabilitation.

Unlike studies that typically report rehabilitation based on absolute changes in FIM scores between delayed and early rehabilitation (28, 31), this study has the advantage of using relative functional gain. For instance, a study reported that patients who were admitted to rehabilitation 31 to 150 days after experiencing stroke tended to show less improvement in FIM scores and lower FIM efficiency compared with those starting rehabilitation earlier (28). However, absolute changes in FIM scores can be misleading as these do not account for the baseline FIM score on rehabilitation admission, which, in turn, influences the patient’s maximum achievable score (32). In contrast, this study used RFG for rehabilitation outcomes, which accounts for patients’ different potentials in achieving FIM score gains relative to their FIM scores on inpatient rehabilitation admission, resulting in a more accurate comparison. Furthermore, to investigate the potential association between delayed inpatient rehabilitation and patients’ speed in regaining lost functions, FIM efficiency was also used in this study. FIM efficiency measures how effectively healthcare resources, such as staff, equipment, and therapy time, are utilized to improve a patient’s functional abilities, quantifying the functional gain per day. By using both relative functional gain and FIM efficiency, this study found a negative association between delayed inpatient rehabilitation and functional gain relative to patients’ maximum potential, as well as a negative association between delayed inpatient rehabilitation and the speed of recovery.

The subgroup analysis in this study further examined the association between delayed inpatient rehabilitation and rehabilitation outcomes across different stroke types. The findings revealed a significant negative association between delayed inpatient rehabilitation and relative functional gain among haemorrhagic stroke patients. This result aligns with previous research, which suggests that early rehabilitation for intracerebral haemorrhage survivors can lead to improved motor recovery, reduced functional and neurological impairments, and an enhanced quality of life (33, 34). In contrast, the negative association between delayed inpatient rehabilitation and relative functional gain was not statistically significant among ischaemic stroke patients or the combined group of ischaemic stroke and stroke of unspecified type. This may suggest that the impact of delayed inpatient rehabilitation on RFG among these groups may be less pronounced. One possible explanation, supported by previous studies, is that patients with haemorrhagic stroke tend to show greater effectiveness in post-rehabilitation functional improvements compared with those with ischaemic stroke, even after controlling for factors such as stroke severity, baseline disability, age, sex, and the time from stroke onset to admission (35). Additionally, this study found that delayed inpatient rehabilitation was significantly associated with reduced FIM efficiency in the combined group of ischaemic stroke and stroke of unspecified type. These findings underscore the importance of timely inpatient rehabilitation, as delays may not only reduce RFG in haemorrhagic stroke patients but also slow the pace of functional recovery in the combined group of patients with ischaemic stroke and stroke of unspecified type. Preventing delays in inpatient rehabilitation is therefore important for optimising functional recovery outcomes across all stroke types.

### Strengths and limitations

By using linked data from the hospital and rehabilitation registry, this study incorporated baseline characteristics at the time of stroke admission and upon admission to rehabilitation into the analysis. This linkage ensured the inclusion of patients transferred to other rehabilitation facilities beyond the participating hospital. Outcome measurements were based on RFG and FIM efficiency rather than absolute changes in FIM scores, accounting for FIM scores on rehabilitation admission and the duration of rehabilitation.

This study has limitations. It analysed only inpatient rehabilitation episodes, excluding those who received rehabilitation in the community. Therefore, the results should be interpreted cautiously when applied to different rehabilitation venues, as inpatient rehabilitation programmes are typically provided to patients with more severe functional losses (36). The rehabilitation outcomes investigated in this study include only RFG and FIM efficiency during inpatient rehabilitation. The overall rehabilitation outcomes for stroke survivors throughout their entire rehabilitation journey were not covered in this study. Restricted by the sample size, comorbidities that could have impacted the rehabilitation progress, but were present in only a small number of patients, were grouped as a single category in the analysis, limiting further exploration. Although stroke severity can be reflected in the FIM scores upon admission to inpatient rehabilitation (37) and was accounted for in the analysis, this study remains limited by the substantial proportion of missing data for stroke severity measurements at the time of acute stroke admission. This cohort study is observational, and causal relationships cannot be inferred. Since the study is not a randomized controlled trial, confounding from other unaccounted factors is always possible.

### Conclusion

More than half of stroke patients experienced delays in starting inpatient rehabilitation after being assessed as ready, predominantly due to the unavailability of rehabilitation beds. Delayed inpatient rehabilitation was associated with poorer outcomes and longer stays in rehabilitation. The results of this study highlight the potential negative impacts of reduced availability of inpatient rehabilitation resources in regional areas and underscore the need for supportive policies within regional health services to address these delays by providing more resources.

## Supplementary Material

DELAYED INPATIENT REHABILITATION AND FUNCTIONAL OUTCOMES FOR ACUTE STROKE: A RETROSPECTIVE COHORT STUDY IN AN AUSTRALIAN REGIONAL HOSPITAL

DELAYED INPATIENT REHABILITATION AND FUNCTIONAL OUTCOMES FOR ACUTE STROKE: A RETROSPECTIVE COHORT STUDY IN AN AUSTRALIAN REGIONAL HOSPITAL

DELAYED INPATIENT REHABILITATION AND FUNCTIONAL OUTCOMES FOR ACUTE STROKE: A RETROSPECTIVE COHORT STUDY IN AN AUSTRALIAN REGIONAL HOSPITAL
