# Supplementary material for: DELAYED INPATIENT REHABILITATION AND FUNCTIONAL OUTCOMES FOR ACUTE STROKE: A RETROSPECTIVE COHORT STUDY IN AN AUSTRALIAN REGIONAL HOSPITAL
Source: J Rehabil Med. 2025 Aug 5;57:42506. doi: 10.2340/jrm.v57.42506 (PMC12340994; doi:10.2340/jrm.v57.42506)
Supplement: DELAYED INPATIENT REHABILITATION AND FUNCTIONAL OUTCOMES FOR ACUTE STROKE: A RETROSPECTIVE COHORT STUDY IN AN AUSTRALIAN REGIONAL HOSPITAL [file JRM-57-42506-s1.pdf]

Supplementary material has been published as submitted. It has not been copyedited, or typeset by Journal of Rehabilitation Medicine

Appendix S1: Description of Function Independence Measure and formulas for calculating Relative Functional Gain

Function Independence Measure consists of 18 items designed to measure a patient's motor and cognitive performance each rated on a 7-point ordinal scale. The total FIM score ranges from 18 to 126. The motor subscale, which includes the first 13 items, has a score range of 13 to 91, while the cognitive subscale, consisting of the last 5 items, ranges from 5 to 35.

The Relative Functional Gain can be calculated using the following formulas:

- If the Discharge FIM is greater than the Admission FIM, then RFG equals (Discharge FIM - Admission FIM) divided by (126 - Admission FIM).
- If the Discharge FIM is less than the Admission FIM, then RFG equals (Discharge FIM - Admission FIM) divided by Admission FIM.
- If the Discharge FIM is equal to the Admission FIM, then RFG is set to 0.

Appendix S2: Description of covariates

Rehabilitation physicians assessed and recorded comorbidities that could impact rehabilitation outcomes including cardiac disease, respiratory disease, drug and alcohol abuse, dementia, mental health conditions, hearing/visual impairment, diabetes mellitus, morbid obesity, arthritis/osteoarthritis/osteoporosis, chronic pain, cancer, and renal failure. Comorbidities that appeared in only a small number of patients were grouped together under the category "other comorbidities". A prior history of stroke was also recorded as a comorbidity in the AROC dataset if pre-existing impairments from a previous stroke were likely to impact the rehabilitation outcome of the current stroke. The patient's comorbidity status was also reflected by the number of comorbidities at the time of their initial acute stroke admission, prior to rehabilitation. The Modified Monash Model (MMM) was used to categorise the remoteness of the patient's residential area (1). Socio-economic status was defined using the Index of Relative Socio-economic Advantage and Disadvantage (IRSAD), based on the patient's residential postcode (2). Covariates from the index acute admission included type of admission ward (stroke unit, intensive care unit (ICU), or coronary care unit (CCU)), admission on a weekday or weekend/holiday; admission time categorised as daytime (8:00 am to 4:00 pm) and night-time (4:00 pm to 8:00 am), and the number of days from stroke onset to rehabilitation admission.

Reference:

1. Department of Health and Aged Care. Modified Monash Model [Internet]. [cited 2023 Dec 23]. Available from: https://www.health.gov.au/topics/rural-healthworkforce/classifications/mmm

2. Australian Bureau of Statistics. Socio-Economic Indexes for Areas [Internet]. 2023 [cited 2023 Dec 23]. Available from: https://www.abs.gov.au/websitedbs/censushome.nsf/home/seifa

Appendix S3: Methods for assessing the fit of mixed-effects linear models and negative binomial regression

To assess the fit of the mixed-effects linear models and to determine whether their assumptions were met, several diagnostic techniques were employed. The congruence between the model and the data was examined by comparing the distribution of values simulated from the fitted model with the distribution of the observed values. Linearity was evaluated by plotting the residuals against the fitted values. The normality of the residuals was investigated using quantile-quantile plots. Furthermore, the normality of the random effects, particularly for the 'Admission FIM motor group,' was also scrutinised through quantile-quantile plots.

Apart from the negative binomial regression, an alternative Poisson regression model for modelling length of stay in rehabilitation was also considered. The presence of overdispersion following fitting Poisson model was demonstrated by the fact that the ratio (5.13) of the residual deviance to the degrees of freedom was greater than one. This finding suggested that the Poisson model was not an adequate fit for the LOS data. Model fitness was also evaluated by comparing the Akaike Information Criterion (AIC) values of both the Poisson model (4451.2) and negative binomial regression model (3366.4). The reduction in the AIC value for the negative binomial model indicates a better fit to the data.

Appendix S4: Variable selection for multivariate mixed effects linear regression for relative functional gain

The Multivariate mixed effects linear regression for relative functional gain was consistently adjusted for age, sex, stroke type, treatment in a stroke unit, admission to ICU or CCU (index admission), the number of comorbidities (index admission), NIHSS (index admission), and patient's ability to walk independently upon index admission. Variables with a $p \leq 0.1$ from univariate models were also adjusted in the multivariate model after examining of potential collinearity based on the pairwise Spearman test. After fitting the multivariate mixed-effects linear regression model for relative functional gain, the VIF values for all variables are below 5 as shown in the following table.

Variable	VIF
Age group (years)	1.55
Sex	1.12
Type of stroke	1.13
Treated in a stroke unit or ICU or CCU	1.88
Walk independently on admission	2.06
NIHSS group	1.61
Number of comorbidities	2.35
Inpatient rehabilitation delayed	1.07
Previous history of stroke	1.12
Existing comorbidity: Cardiac disease	1.11
Existing comorbidity: Cancer	1.09
Existing comorbidity: Dementia	1.11
Existing comorbidity: Other	1.08
Experienced complications during rehabilitation	1.39
Employment status prior to stroke	1.12
From onset of stroke to inpatient rehabilitation (scaled days)	1.18
FIM total score on rehabilitation admission (scaled)	2.66

Appendix S5: Variable selection for multivariate mixed effects linear regression for Functional Independence Measure efficiency

The Multivariate mixed effects linear regression for Functional Independence Measure efficiency was consistently adjusted for age, sex, stroke type, treatment in a stroke unit, admission to ICU or CCU (index admission), the number of comorbidities (index admission), NIHSS (index admission), and patient's ability to walk independently upon index admission. Variables with a $p \leq 0.1$ from univariate models were also adjusted in the multivariate model after examining of potential collinearity based on the pairwise Spearman test. After fitting the multivariate mixed-effects linear regression model for Functional Independence Measure efficiency, the VIF values for all variables are below 5 as shown in the following table.

Variable	VIF
Age group (years)	1.51
Sex	1.13
Treated in a stroke unit or ICU or CCU	1.83
Type of stroke	1.13
Number of comorbidities	1.06
Walk independently on admission	1.99
NIHSS group	1.61
Inpatient rehabilitation delayed	2.19
Existing comorbidity: Cardiac disease	1.08
Previous history of stroke	1.11
Experienced complications during rehabilitation	1.36
From onset of stroke to inpatient rehabilitation (scaled days)	1.11
FIM total score on rehabilitation admission (scaled)	1.18
Employment status prior to stroke	2.41

Appendix S6: Variable selection for multivariate negative binomial regression for length of stay in rehabilitation

The multivariate negative binomial regression for length of stay in rehabilitation was always adjusted for age, sex, stroke type, treatment in a stroke unit, admission to ICU or CCU (index admission), and number of comorbidities (index admission) NIHSS (index admission), patient's ability to walk independently upon index admission. Variables with a $p \leq 0.1$ from univariate models in the negative binomial regressions were also adjusted in the multivariate regression after the assessment of potential collinearity based on the pairwise Spearman test. After fitting the multivariate negative binomial regression for length of stay in rehabilitation, the VIF values for all variables are below 5 as shown in the following table.

Variable	VIF
Age group (years)	1.48
Sex	1.16
Type of stroke	1.13
Treated in a stroke unit or ICU or CCU	1.84
NIHSS group	1.68
Walk independently on admission	2.04
Number of comorbidities	1.14
Admitted in weekday or non weekday	1.05
Inpatient rehabilitation delayed	2.25
Previous history of stroke	1.14
Existing comorbidity: Dementia	1.09
Existing comorbidity: Hearing/visual impairment	1.06
From onset of stroke to inpatient rehabilitation (scaled days)	1.2
Experienced complications during rehabilitation	1.43
Employment status prior to stroke	2.47
FIM total score on rehabilitation admission (scaled)	1.38
