# Supplementary material for: DELAYED INPATIENT REHABILITATION AND FUNCTIONAL OUTCOMES FOR ACUTE STROKE: A RETROSPECTIVE COHORT STUDY IN AN AUSTRALIAN REGIONAL HOSPITAL
Source: J Rehabil Med. 2025 Aug 5;57:42506. doi: 10.2340/jrm.v57.42506 (PMC12340994; doi:10.2340/jrm.v57.42506)
Supplement: DELAYED INPATIENT REHABILITATION AND FUNCTIONAL OUTCOMES FOR ACUTE STROKE: A RETROSPECTIVE COHORT STUDY IN AN AUSTRALIAN REGIONAL HOSPITAL [file JRM-57-42506-s2.pdf]

Supplementary material has been published as submitted. It has not been copyedited, or typeset by Journal of Rehabilitation Medicine

Table SI. Patients' characteristics on stroke and rehabilitation admission

Variable	N = 584[1]
**Age (years)***	76 (67.0, 83.0)
**Sex***	
Male	325 (55.7%)
Female	259 (44.3%)
**Type of stroke***	
Haemorrhagic stroke	69 (11.8%)
Ischaemic stroke	474 (81.2%)
Stroke of unspecified type	41 (7.0%)
**Treated in a stroke unit or ICU or CCU***	
No	374 (64.0%)
Yes	210 (36.0%)
**Number of comorbidities***	2 (2.0, 3.0)
**Country of birth***	
Australia	252 (43.2%)
Overseas	33 (5.7%)
Unknown	299 (51.2%)
**Social economic Status***	
Below median	357 (61.3%)
Median and above	225 (38.7%)
**Walk independently on admission***	
Yes	34 (5.8%)
No	250 (42.8%)
Unknown	300 (51.4%)
**NIHSS group***	
Mild	45 (7.7%)
Moderate	54 (9.2%)
Severe	15 (2.6%)
Unknown	470 (80.5%)
**Indigenous background***	
No	577 (99.0%)
Yes	6 (1.0%)
**Modified Monash Model remoteness***	
Metropolitan area/regional centre	354 (60.8%)
Rural area	228 (39.2%)
**Admitted in daytime or night-time***	
Daytime	239 (40.9%)
Nighttime	345 (59.1%)
**Admitted in weekday or non weekday***	
Weekday	448 (76.7%)
Weekend/Holiday	136 (23.3%)
**Inpatient rehabilitation delayed^**	
No	186 (31.8%)
Yes	301 (51.5%)
Unknown	97 (16.6%)
**Days delayed in starting inpatient rehabilitation^**	2 (1.0, 4.0)
**Existing comorbidity: Cardiac disease^**	
No	466 (79.8%)
Yes	118 (20.2%)
**Existing comorbidity: Respiratory disease^**	
No	541 (92.6%)
Yes	43 (7.4%)
**Existing comorbidity: Drug and Alcohol abuse^**	
No	567 (97.1%)
Yes	17 (2.9%)
**Existing comorbidity: Mental health problem^**	
No	540 (92.5%)
Yes	44 (7.5%)
**Previous history of stroke^**	
No	522 (89.4%)
Yes	62 (10.6%)
**Existing comorbidity: Diabetes mellites^**	
No	514 (88.0%)

Table SI. Patients' characteristics on stroke and rehabilitation admission	
**Variable**	**N = 584**[1]
Yes	70 (12.0%)
**Existing comorbidity: Morbid obesity^**	
No	570 (97.6%)
Yes	14 (2.4%)
**Existing comorbidity: Chronic pain^**	
No	568 (97.3%)
Yes	16 (2.7%)
**Existing comorbidity: Cancer^**	
No	561 (96.1%)
Yes	23 (3.9%)
**Existing comorbidity: Dementia^**	
No	562 (96.2%)
Yes	22 (3.8%)
**Existing comorbidity: Renal failure^**	
No	573 (98.1%)
Yes	11 (1.9%)
**Existing comorbidity: Arthritis/osteoarthritis/osteoporosis^**	
No	509 (87.2%)
Yes	75 (12.8%)
**Existing comorbidity: Hearing/visual impairment^**	
No	558 (95.5%)
Yes	26 (4.5%)
**Existing comorbidity: Other^**	
No	490 (83.9%)
Yes	94 (16.1%)
**From onset of stroke to inpatient rehabilitation (days)^**	9 (6.0, 14.0)
**Employment status prior to stroke**	
Retired	371 (63.5%)
Employed	93 (15.9%)
Not employed	120 (20.5%)
**Experienced complications during rehabilitation**	
No	452 (77.4%)
Unknown	7 (1.2%)
Yes	125 (21.4%)
**FIM total score^**	75 (51.0, 93.0)
**FIM motor score^**	54 (33.0, 68.0)
**FIM cognition score^**	22 (15.0, 27.0)

[1]Median (IQR); n (%)

**On stroke admission*

^*On rehabilitation admission*

Table SII. Multivariate mixed effects linear regression for relative functional gain (Inpatient rehabilitation delayed)			
**Characteristic**	**Beta**	**95% CI[1]**	**p-value**
**Age group (years)***			
Under 75	—	—	
75-84	-0.07	-0.13, -0.02	0.009
85 or more	-0.16	-0.22, -0.09	<0.001
**Sex***			
Male	—	—	
Female	-0.02	-0.06, 0.03	0.517
**Type of stroke***			
Stroke of unspecified type	—	—	
Ischaemic stroke	-0.03	-0.12, 0.05	0.454
Haemorrhagic stroke	0.04	-0.07, 0.14	0.509
**Treated in a stroke unit or ICU or CCU***			
No	—	—	
Yes	0.05	-0.01, 0.11	0.085
**Walk independently on admission***			
Yes	—	—	
No	0.11	0.02, 0.21	0.023
Unknown	0.15	0.04, 0.25	0.007
**NIHSS group***			
mild	—	—	
moderate	-0.03	-0.13, 0.08	0.609
severe	-0.04	-0.20, 0.12	0.608
Unknown	-0.06	-0.15, 0.03	0.165
**Inpatient rehabilitation delayed^**			
No	—	—	
Yes	-0.07	-0.11, -0.02	0.009
Unknown	0.03	-0.07, 0.14	0.546
**Number of comorbidities***	-0.01	-0.04, 0.01	0.362
**Previous history of stroke^**			
No	—	—	
Yes	0.00	-0.08, 0.08	0.969
**Existing comorbidity: Cardiac disease^**			
No	—	—	
Yes	-0.04	-0.10, 0.02	0.153
**Existing comorbidity: Cancer^**			
No	—	—	
Yes	-0.04	-0.15, 0.07	0.472
**Existing comorbidity: Dementia^**			
No	—	—	
Yes	-0.12	-0.24, 0.00	0.052
**Existing comorbidity: Other^**			
No	—	—	
Yes	-0.06	-0.12, 0.00	0.038
**Experienced complications during rehabilitation**			
No	—	—	
Yes	-0.09	-0.15, -0.03	0.003
Unknown	-0.28	-0.48, -0.07	0.009
**From onset of stroke to inpatient rehabilitation (scaled days)^**	-0.04	-0.07, -0.02	<0.001
**FIM total score on rehabilitation admission (scaled)^**	0.09	0.04, 0.13	<0.001
**Employment status prior to stroke**			
Retired	—	—	
Employed	0.03	-0.04, 0.10	0.379
Not employed	0.00	-0.09, 0.08	0.916

[1]CI = Confidence Interval

**On stroke admission*

^*On rehabilitation admission*

Table SIII. Multivariate mixed effects linear regression for Functional Independence Measure efficiency (Inpatient rehabilitation delayed)

Characteristic	Beta	95% CI[1]	p-value
**Age group (years)***			
Under 75	—	—	
75-84	-0.01	-0.17, 0.16	0.946
85 or more	-0.21	-0.41, -0.02	0.029
**Sex***			
Male	—	—	
Female	-0.08	-0.21, 0.06	0.281
**Treated in a stroke unit or ICU or CCU***			
No	—	—	
Yes	0.06	-0.12, 0.24	0.492
**Type of stroke***			
Stroke of unspecified type	—	—	
Ischaemic stroke	-0.24	-0.49, 0.02	0.068
Haemorrhagic stroke	0.04	-0.27, 0.35	0.813
**Number of comorbidities***	-0.06	-0.14, 0.02	0.151
**Walk independently on admission***			
Yes	—	—	
No	0.13	-0.15, 0.41	0.355
Unknown	0.28	-0.03, 0.58	0.080
**NIHSS group***			
mild	—	—	
moderate	0.01	-0.29, 0.31	0.966
severe	-0.13	-0.61, 0.35	0.587
Unknown	-0.32	-0.57, -0.07	0.013
**Inpatient rehabilitation delayed^**			
No	—	—	
Yes	-0.18	-0.32, -0.04	0.014
Unknown	0.24	-0.08, 0.56	0.147
**Existing comorbidity: Cardiac disease^**			
No	—	—	
Yes	-0.11	-0.29, 0.06	0.199
**Previous history of stroke^**			
No	—	—	
Yes	-0.09	-0.32, 0.14	0.456
**Experienced complications during rehabilitation**			
No	—	—	
Yes	-0.42	-0.60, -0.24	<0.001
Unknown	-1.0	-1.6, -0.42	<0.001
**From onset of stroke to inpatient rehabilitation (scaled days)^**	-0.12	-0.21, -0.03	0.006
**FIM total score on rehabilitation admission (scaled)^**	-0.50	-0.65, -0.34	<0.001
**Employment status prior to stroke**			
Retired	—	—	
Employed	0.19	-0.01, 0.40	0.066
Not employed	-0.15	-0.40, 0.09	0.211

[1]CI = Confidence Interval

**On stroke admission*

^*On rehabilitation admission*

Table SIV. Negative binomial regression for length of stay in rehabilitation (Inpatient rehabilitation delayed)			
**Characteristic**	**IRR[1]**	**95% CI[1]**	**p-value**
**Age group (years)***			
Under 75	—	—	
75-84	0.91	0.82, 1.01	0.073
85 or more	0.82	0.73, 0.93	0.001
**Sex***			
Male	—	—	
Female	1.01	0.92, 1.10	0.883
**Type of stroke***			
Stroke of unspecified type	—	—	
Ischaemic stroke	1.24	1.05, 1.46	0.009
Haemorrhagic stroke	1.13	0.93, 1.37	0.228
**Treated in a stroke unit or ICU or CCU***			
No	—	—	
Yes	1.05	0.94, 1.17	0.385
**NIHSS group***			
mild	—	—	
moderate	1.12	0.93, 1.36	0.238
severe	1.01	0.76, 1.36	0.925
Unknown	1.28	1.09, 1.51	0.002
**Walk independently on admission***			
Yes	—	—	
No	0.93	0.78, 1.11	0.454
Unknown	1.00	0.83, 1.22	0.973
**Number of comorbidities***	0.99	0.94, 1.04	0.588
**Admitted in weekday or non weekday***			
Business day	—	—	
Weekend/Holiday	0.99	0.90, 1.09	0.812
**Inpatient rehabilitation delayed^**			
No	—	—	
Yes	1.11	1.02, 1.21	0.021
Unknown	1.01	0.83, 1.23	0.923
**Previous history of stroke^**			
No	—	—	
Yes	1.08	0.94, 1.25	0.287
**Existing comorbidity: Dementia^**			
No	—	—	
Yes	0.61	0.49, 0.76	<0.001
**Existing comorbidity: Hearing/visual impairment^**			
No	—	—	
Yes	0.83	0.68, 1.01	0.053
**From onset of stroke to inpatient rehabilitation (scaled days)^**	1.00	0.94, 1.05	0.864
**Experienced complications during rehabilitation**			
No	—	—	
Yes	1.28	1.15, 1.43	<0.001
Unknown	1.00	0.70, 1.46	0.985
**Employment status prior to stroke**			
Employed	—	—	
Not employed	1.16	0.98, 1.37	0.076
Retired	1.13	1.00, 1.29	0.056
**FIM total score on rehabilitation admission (scaled)^**	0.63	0.60, 0.66	<0.001

[1]IRR = Incidence Rate Ratio, CI = Confidence Interval

**On stroke admission*

^*On rehabilitation admission*

Table SV. Multivariate mixed effects linear regression for RFG (sensitivity analysis)			
**Characteristic**	**Beta**	**95% CI[1]**	**p-value**
**Age group (years)***			
Under 75	—	—	
75-84	-0.07	-0.13, -0.02	0.013
85 or more	-0.16	-0.23, -0.09	<0.001
**Sex***			
Male	—	—	
Female	-0.01	-0.06, 0.04	0.705
**Type of stroke***			
Stroke of unspecified type	—	—	
Ischaemic stroke	-0.03	-0.12, 0.06	0.506
Haemorrhagic stroke	0.05	-0.06, 0.16	0.367
**Treated in a stroke unit or ICU or CCU***			
No	—	—	
Yes	0.05	-0.02, 0.11	0.147
**Walk independently on admission***			
Yes	—	—	
No	0.09	-0.01, 0.19	0.075
Unknown	0.13	0.02, 0.24	0.017
**NIHSS group***			
Mild	—	—	
Moderate	-0.04	-0.15, 0.07	0.515
Severe	-0.03	-0.20, 0.14	0.759
Unknown	-0.07	-0.16, 0.02	0.131
**Inpatient rehabilitation delayed^**			
No	—	—	
Yes	-0.07	-0.12, -0.01	0.012
Unknown	0.03	-0.08, 0.14	0.563
**Number of comorbidities***	-0.02	-0.04, 0.01	0.295
**Previous history of stroke^**			
No	—	—	
Yes	0.00	-0.08, 0.08	0.936
**Existing comorbidity: Cardiac disease^**			
No	—	—	
Yes	-0.05	-0.11, 0.01	0.098
**Existing comorbidity: Cancer^**			
No	—	—	
Yes	-0.05	-0.17, 0.06	0.370
**Existing comorbidity: Dementia^**			
No	—	—	
Yes	-0.12	-0.25, 0.01	0.065
**Existing comorbidity: Other^**			
No	—	—	
Yes	-0.05	-0.11, 0.01	0.111
**Experienced complications during rehabilitation**			
No	—	—	
Yes	-0.09	-0.16, -0.03	0.002
Unknown	-0.28	-0.49, -0.07	0.010
**From onset of stroke to rehabilitation (scaled days)^**	-0.04	-0.07, -0.02	0.002
**FIM total score on rehabilitation admission (scaled)^**	0.08	0.03, 0.13	0.003
**Employment status prior to stroke**			
Retired	—	—	
Employed	0.03	-0.05, 0.10	0.462
Not employed	-0.01	-0.10, 0.08	0.832

[1]CI = Confidence Interval

**On stroke admission*

^*On rehabilitation admission*

**Table SVI. Multivariate mixed effects linear regression for FIM efficiency (sensitivity analysis)**			
**Characteristic**	**Beta**	**95% CI[1]**	**p-value**
**Age group (years)***			
Under 75	—	—	
75-84	-0.01	-0.18, 0.16	0.938
85 or more	-0.21	-0.41, -0.01	0.036
**Sex***			
Male	—	—	
Female	-0.09	-0.23, 0.05	0.229
**Treated in a stroke unit or ICU or CCU***			
No	—	—	
Yes	0.04	-0.15, 0.22	0.676
**Type of stroke***			
Stroke of unspecified type	—	—	
Ischaemic stroke	-0.22	-0.48, 0.04	0.095
Haemorrhagic stroke	0.07	-0.25, 0.38	0.669
**Number of comorbidities***	-0.06	-0.15, 0.02	0.136
**Walk independently on admission***			
Yes	—	—	
No	0.13	-0.15, 0.41	0.359
Unknown	0.29	-0.02, 0.60	0.069
**NIHSS group***			
Mild	—	—	
Moderate	-0.05	-0.36, 0.26	0.738
Severe	-0.18	-0.66, 0.31	0.469
Unknown	-0.38	-0.64, -0.12	0.004
**Inpatient rehabilitation delayed^**			
No	—	—	
Yes	-0.17	-0.31, -0.03	0.020
Unknown	0.21	-0.12, 0.54	0.205
**Existing comorbidity: Cardiac disease^**			
No	—	—	
Yes	-0.13	-0.31, 0.05	0.160
**Previous history of stroke^**			
No	—	—	
Yes	-0.09	-0.33, 0.15	0.460
**Experienced complications during rehabilitation**			
No	—	—	
Yes	-0.40	-0.59, -0.22	<0.001
Unknown	-0.99	-1.6, -0.41	<0.001
**From onset of stroke to rehabilitation (scaled days)^**	-0.10	-0.19, -0.02	0.022
**FIM total score on rehabilitation admission (scaled)^**	-0.53	-0.69, -0.36	<0.001
**Employment status prior to stroke**			
Retired	—	—	
Employed	0.21	0.00, 0.42	0.052
Not employed	-0.14	-0.38, 0.11	0.287

[1]CI = Confidence Interval

**On stroke admission*

^*On rehabilitation admission*

Table SVII. Negative binomial regression for length of stay in rehabilitation (sensitivity analysis)			
**Characteristic**	**IRR**[1]	**95% CI**[1]	**p-value**
**Age group (years)***			
Under 75	—	—	
75-84	0.91	0.82, 1.01	0.086
85 or more	0.83	0.73, 0.93	0.002
**Sex***			
Male	—	—	
Female	1.02	0.93, 1.11	0.653
**Type of stroke***			
Stroke of unspecified type	—	—	
Ischaemic stroke	1.22	1.03, 1.43	0.020
Haemorrhagic stroke	1.09	0.90, 1.33	0.383
**Treated in a stroke unit or ICU or CCU***			
No	—	—	
Yes	1.05	0.94, 1.17	0.400
**NIHSS group***			
Mild	—	—	
Moderate	1.11	0.92, 1.35	0.284
Severe	1.00	0.75, 1.34	0.998
Unknown	1.30	1.10, 1.53	0.002
**Walk independently on admission***			
Yes	—	—	
No	0.93	0.78, 1.11	0.417
Unknown	0.99	0.82, 1.21	0.949
**Number of comorbidities***	0.98	0.93, 1.04	0.539
**Admitted in weekday or non weekday***			
Business day	—	—	
Weekend Holiday	0.99	0.90, 1.09	0.863
**Inpatient rehabilitation delayed^**			
No	—	—	
Yes	1.12	1.03, 1.22	0.012
Unknown	1.01	0.83, 1.23	0.947
**Previous history of stroke^**			
No	—	—	
Yes	1.06	0.92, 1.23	0.438
**Existing comorbidity: Dementia^**			
No	—	—	
Yes	0.63	0.51, 0.79	<0.001
**Existing comorbidity: Hearing/visual impairment^**			
No	—	—	
Yes	0.82	0.68, 1.00	0.049
**From onset of stroke to rehabilitation (scaled days)^**	1.00	0.94, 1.05	0.890
**Experienced complications during rehabilitation**			
No	—	—	
Yes	1.28	1.15, 1.43	<0.001
Unknown	1.01	0.71, 1.47	0.948
**Employment status prior to stroke**			
Employed	—	—	
Not employed	1.18	1.00, 1.39	0.052
Retired	1.14	1.00, 1.30	0.044
**FIM total score on rehabilitation admission (scaled)^**	0.62	0.59, 0.65	<0.001

[1]IRR = Incidence Rate Ratio, CI = Confidence Interval

**On stroke admission*

^*On rehabilitation admission*

Table SVIII. Multivariate mixed effects linear regression for RFG (Ischaemic stroke)			
**Characteristic**	**Beta**	**95% CI[1]**	**p-value**
**Age group (years)***			
Under 75	—	—	
75-84	-0.07	-0.13, 0.00	0.048
85 or more	-0.14	-0.22, -0.07	<0.001
**Sex***			
Male	—	—	
Female	-0.02	-0.07, 0.04	0.535
**Treated in a stroke unit or ICU or CCU***			
No	—	—	
Yes	0.06	-0.01, 0.13	0.091
**Number of comorbidities***	-0.01	-0.04, 0.02	0.499
**Walk independently on admission***			
Yes	—	—	
No	0.12	0.02, 0.23	0.025
Unknown	0.16	0.04, 0.28	0.011
**NIHSS group***			
mild	—	—	
moderate	-0.01	-0.13, 0.10	0.815
severe	-0.02	-0.19, 0.14	0.775
Unknown	-0.06	-0.16, 0.04	0.224
**Inpatient rehabilitation delayed^**			
No	—	—	
Yes	-0.05	-0.11, 0.00	0.072
Unknown	0.06	-0.06, 0.19	0.329
**Existing comorbidity: Cardiac disease^**			
No	—	—	
Yes	-0.04	-0.11, 0.03	0.221
**Previous history of stroke^**			
No	—	—	
Yes	-0.02	-0.11, 0.07	0.737
**Existing comorbidity: Cancer^**			
No	—	—	
Yes	-0.05	-0.17, 0.07	0.401
**Existing comorbidity: Dementia^**			
No	—	—	
Yes	-0.10	-0.25, 0.05	0.176
**Existing comorbidity: Other^**			
No	—	—	
Yes	-0.05	-0.12, 0.02	0.167
**From onset of stroke to inpatient rehabilitation (scaled days)^**	-0.04	-0.07, -0.02	0.002
**Employment status prior to stroke**			
Retired	—	—	
Employed	0.04	-0.05, 0.12	0.391
Not employed	-0.02	-0.11, 0.08	0.754
**Experienced complications during rehabilitation**			
No	—	—	
Yes	-0.11	-0.18, -0.05	<0.001
Unknown	-0.27	-0.53, -0.02	0.033
**FIM total score on rehabilitation admission (scaled)^**	0.09	0.04, 0.14	0.004

[1]CI = Confidence Interval

**On stroke admission*

^*On rehabilitation admission*

Table SIX. Multivariate mixed effects linear regression for RFG (Ischaemic stroke and stroke of unspecified type)			
**Characteristic**	**Beta**	**95% CI[1]**	**p-value**
**Age group (years)***			
Under 75	—	—	
75-84	-0.08	-0.14, -0.02	0.015
85 or more	-0.17	-0.24, -0.10	<0.001
**Sex***			
Male	—	—	
Female	-0.02	-0.07, 0.03	0.495
**Treated in a stroke unit or ICU or CCU***			
No	—	—	
Yes	0.06	-0.01, 0.12	0.087
**Number of comorbidities***	-0.01	-0.04, 0.02	0.480
**Walk independently on admission***			
Yes	—	—	
No	0.11	0.01, 0.21	0.037
Unknown	0.15	0.04, 0.27	0.007
**NIHSS group***			
mild	—	—	
moderate	-0.01	-0.12, 0.10	0.824
severe	-0.02	-0.19, 0.15	0.812
Unknown	-0.06	-0.16, 0.03	0.207
**Inpatient rehabilitation delayed^**			
No	—	—	
Yes	-0.05	-0.10, 0.00	0.063
Unknown	0.05	-0.07, 0.17	0.417
**Existing comorbidity: Cardiac disease^**			
No	—	—	
Yes	-0.03	-0.10, 0.03	0.315
**Previous history of stroke^**			
No	—	—	
Yes	-0.01	-0.10, 0.08	0.811
**Existing comorbidity: Cancer^**			
No	—	—	
Yes	-0.04	-0.16, 0.07	0.453
**Existing comorbidity: Dementia^**			
No	—	—	
Yes	-0.12	-0.26, 0.01	0.063
**Existing comorbidity: Other^**			
No	—	—	
Yes	-0.06	-0.12, 0.01	0.094
**From onset of stroke to inpatient rehabilitation (scaled days)^**	-0.05	-0.07, -0.02	<0.001
**Employment status prior to stroke**			
Retired	—	—	
Employed	0.01	-0.07, 0.09	0.757
Not employed	-0.02	-0.11, 0.07	0.663
**Experienced complications during rehabilitation**			
No	—	—	
Yes	-0.11	-0.18, -0.05	<0.001
Unknown	-0.23	-0.45, 0.00	0.052
**FIM total score on rehabilitation admission (scaled)^**	0.09	0.04, 0.14	0.002

[1]CI = Confidence Interval

**On stroke admission*

^*On rehabilitation admission*

Table SX. Multivariate mixed effects linear regression for RFG (Haemorrhagic stroke)			
**Characteristic**	**Beta**	**95% CI[1]**	**p-value**
**Age group (years)***			
Under 75	—	—	
75-84	-0.10	-0.24, 0.03	0.128
85 or more	-0.09	-0.25, 0.08	0.295
**Sex***			
Male	—	—	
Female	0.03	-0.09, 0.15	0.637
**Treated in a stroke unit or ICU or CCU***			
No	—	—	
Yes	-0.03	-0.18, 0.11	0.629
**Number of comorbidities***	-0.06	-0.13, 0.02	0.142
**Walk independently on admission***			
Yes	—	—	
No	0.08	-0.24, 0.40	0.617
Unknown	0.02	-0.31, 0.35	0.907
**Social economic Status***			
Below median	—	—	
Median and above	0.11	-0.01, 0.22	0.078
**Inpatient rehabilitation delayed^**			
No	—	—	
Yes	-0.15	-0.28, -0.02	0.025
Unknown	-0.05	-0.25, 0.15	0.605
**Existing comorbidity: Cardiac disease^**			
No	—	—	
Yes	-0.09	-0.23, 0.05	0.186
**Existing comorbidity: Morbid obesity^**			
No	—	—	
Yes	-0.33	-0.66, 0.01	0.054
**Existing comorbidity: Dementia^**			
No	—	—	
Yes	0.01	-0.30, 0.31	0.972
**From onset of stroke to inpatient rehabilitation (scaled days)^**	-0.06	-0.11, -0.01	0.014
**Experienced complications during rehabilitation**			
No	—	—	
Yes	0.16	-0.01, 0.32	0.059
Unknown	-0.52	-1.0, -0.03	0.039
**FIM total score on rehabilitation admission (scaled)^**	0.12	0.00, 0.24	0.045

[1]CI = Confidence Interval

**On stroke admission*

^*On rehabilitation admission*

Table SXI. Multivariate mixed effects linear regression for FIM efficiency (Ischaemic stroke)			
**Characteristic**	**Beta**	**95% CI[1]**	**p-value**
**Age group (years)***			
Under 75	—	—	
75-84	0.01	-0.19, 0.20	0.955
85 or more	-0.23	-0.45, -0.01	0.042
**Sex***			
Male	—	—	
Female	-0.03	-0.19, 0.13	0.712
**Treated in a stroke unit or ICU or CCU***			
No	—	—	
Yes	0.08	-0.11, 0.28	0.404
**Number of comorbidities***	-0.07	-0.16, 0.02	0.133
**Walk independently on admission***			
Yes	—	—	
No	0.09	-0.22, 0.40	0.578
Unknown	0.25	-0.10, 0.60	0.159
**NIHSS group***			
mild	—	—	
moderate	0.09	-0.23, 0.41	0.582
severe	-0.04	-0.53, 0.45	0.878
Unknown	-0.24	-0.51, 0.04	0.092
**Inpatient rehabilitation delayed^**			
No	—	—	
Yes	-0.14	-0.30, 0.01	0.073
Unknown	0.44	0.07, 0.81	0.019
**Existing comorbidity: Cardiac disease^**			
No	—	—	
Yes	-0.19	-0.39, 0.01	0.058
**Previous history of stroke^**			
No	—	—	
Yes	-0.21	-0.48, 0.06	0.122
**From onset of stroke to inpatient rehabilitation (scaled days)^**	-0.11	-0.20, -0.01	0.032
**Employment status prior to stroke**			
Retired	—	—	
Employed	0.18	-0.05, 0.41	0.118
Not employed	-0.19	-0.46, 0.09	0.184
**Experienced complications during rehabilitation**			
No	—	—	
Yes	-0.43	-0.63, -0.23	<0.001
Unknown	-1.3	-1.9, -0.58	<0.001
**FIM total score on rehabilitation admission (scaled)^**	-0.52	-0.70, -0.35	<0.001

[1]CI = Confidence Interval

**On stroke admission*

^*On rehabilitation admission*

**Table SXII. Multivariate mixed effects linear regression for FIM efficiency (Haemorrhagic stroke)**			
**Characteristic**	**Beta**	**95% CI[1]**	**p-value**
**Age group (years)***			
Under 75	—	—	
75-84	0.08	-0.40, 0.57	0.734
85 or more	0.41	-0.16, 0.99	0.153
**Sex***			
Male	—	—	
Female	-0.38	-0.82, 0.06	0.090
**Treated in a stroke unit or ICU or CCU***			
No	—	—	
Yes	-0.13	-0.66, 0.41	0.639
**Number of comorbidities***	-0.10	-0.36, 0.16	0.430
**Walk independently on admission***			
Yes	—	—	
No	0.00	-1.2, 1.2	0.999
Unknown	-0.02	-1.2, 1.2	0.977
**NIHSS group***			
mild	—	—	
moderate	-0.68	-2.4, 1.0	0.424
Unknown	-0.89	-1.7, -0.10	0.027
**Inpatient rehabilitation delayed^**			
No	—	—	
Yes	-0.43	-0.92, 0.06	0.087
Unknown	-0.40	-1.0, 0.21	0.190

[1]CI = Confidence Interval

**On stroke admission*

^*On rehabilitation admission*

**Table SXIII. Multivariate mixed effects linear regression for FIM efficiency (Ischaemic stroke and Stroke of unspecified type)**			
**Characteristic**	**Beta**	**95% CI[1]**	**p-value**
**Age group (years)***			
Under 75	—	—	
75-84	0.02	-0.16, 0.20	0.827
85 or more	-0.23	-0.44, -0.03	0.025
**Sex***			
Male	—	—	
Female	-0.03	-0.18, 0.12	0.693
**Treated in a stroke unit or ICU or CCU***			
No	—	—	
Yes	0.08	-0.11, 0.27	0.424
**Number of comorbidities***	-0.05	-0.13, 0.04	0.261
**Walk independently on admission***			
Yes	—	—	
No	0.08	-0.21, 0.37	0.577
Unknown	0.28	-0.05, 0.60	0.096
**NIHSS group***			
mild	—	—	
moderate	0.13	-0.18, 0.44	0.402
severe	-0.04	-0.53, 0.44	0.856
Unknown	-0.24	-0.50, 0.03	0.082
**Inpatient rehabilitation delayed^**			
No	—	—	
Yes	-0.16	-0.30, -0.01	0.039
Unknown	0.36	0.02, 0.71	0.041
**Existing comorbidity: Cardiac disease^**			
No	—	—	
Yes	-0.15	-0.33, 0.04	0.126
**Experienced complications during rehabilitation**			
No	—	—	
Yes	-0.43	-0.62, -0.24	<0.001
Unknown	-1.0	-1.7, -0.41	0.001
**FIM total score on rehabilitation admission (scaled)^**	-0.47	-0.64, -0.31	<0.001
**Previous history of stroke^**			
No	—	—	
Yes	-0.19	-0.45, 0.08	0.162
**From onset of stroke to inpatient rehabilitation (scaled days)^**	-0.11	-0.21, -0.02	0.015
**Employment status prior to stroke**			
Retired	—	—	
Employed	0.16	-0.05, 0.38	0.142
Not employed	-0.18	-0.44, 0.08	0.170

[1]CI = Confidence Interval

**On stroke admission*

^*On rehabilitation admission*

Table SXIV. Univariate mixed effects linear regression for relative functional gain				
**Characteristic**	**N**	**Beta**	**95% CI**[1]	**p-value**
**Age group (years)***	582			
Under 75		—	—	
75-84		-0.11	-0.16, -0.06	<0.001
85 or more		-0.19	-0.24, -0.13	<0.001
**Sex***	582			
Male		—	—	
Female		-0.04	-0.09, 0.00	0.070
**Type of stroke***	582			
Stroke of unspecified type		—	—	
Ischaemic stroke		0.00	-0.09, 0.08	0.914
Haemorrhagic stroke		0.06	-0.05, 0.17	0.259
**Treated in a stroke unit or ICU or CCU***	582			
No		—	—	
Yes		0.02	-0.03, 0.06	0.462
**Number of comorbidities***	582	-0.03	-0.05, 0.00	0.068
**Country of birth***	582			
Australia		—	—	
Foreign countries		0.04	-0.06, 0.14	0.480
Unknown		0.03	-0.02, 0.07	0.256
**Social economic Status***	580			
Below median		—	—	
Median and above		0.02	-0.03, 0.06	0.500
**Walk independently on admission***	582			
Yes		—	—	
No		0.05	-0.05, 0.15	0.329
Unknown		0.07	-0.03, 0.17	0.194
**NIHSS group***	582			
mild		—	—	
moderate		-0.02	-0.13, 0.09	0.719
severe		-0.06	-0.23, 0.11	0.487
Unknown		-0.05	-0.14, 0.04	0.258
**Indigenous status***	581			
No		—	—	
Yes		-0.17	-0.40, 0.05	0.127
**Modified Monash Model remoteness***	580			
Metropolitan area/regional centre		—	—	
Rural area		0.03	-0.01, 0.08	0.183
**Admitted in daytime or night-time***	582			
Daytime		—	—	
Nighttime		0.00	-0.04, 0.05	0.850
**Admitted in weekday or non weekday***	582			
Business day		—	—	
Weekend or Holiday		0.03	-0.02, 0.09	0.210
**Inpatient rehabilitation delayed^**	582			
No		—	—	
Yes		-0.06	-0.11, -0.01	0.022
Unknown		0.01	-0.06, 0.08	0.848
**Days delayed in starting inpatient rehabilitation^**	483	-0.02	-0.02, -0.01	<0.001
**Existing comorbidity: Cardiac disease^**	582			
No		—	—	
Yes		-0.07	-0.13, -0.01	0.015
**Existing comorbidity: Respiratory disease^**	582			
No		—	—	
Yes		-0.03	-0.12, 0.06	0.504
**Existing comorbidity: Drug and Alcohol abuse^**	582			
No		—	—	
Yes		-0.10	-0.23, 0.03	0.146
**Existing comorbidity: Mental health problem^**	582			
No		—	—	
Yes		-0.04	-0.12, 0.05	0.384
**Previous history of stroke^**	582			
No		—	—	

Table SXIV. Univariate mixed effects linear regression for relative functional gain				
**Characteristic**	**N**	**Beta**	**95% CI[1]**	**p-value**
Yes		-0.08	-0.15, 0.00	0.043
**Existing comorbidity: Diabetes mellites^**	582			
No		—	—	
Yes		-0.05	-0.12, 0.02	0.183
**Existing comorbidity: Morbid obesity^**	582			
No		—	—	
Yes		-0.08	-0.23, 0.06	0.267
**Existing comorbidity: Chronic pain^**	582			
No		—	—	
Yes		-0.02	-0.16, 0.12	0.758
**Existing comorbidity: Cancer^**	582			
No		—	—	
Yes		-0.10	-0.22, 0.01	0.087
**Existing comorbidity: Dementia^**	582			
No		—	—	
Yes		-0.19	-0.31, -0.07	0.002
**Existing comorbidity: Renal failure^**	582			
No		—	—	
Yes		-0.05	-0.21, 0.12	0.573
**Existing comorbidity: Arthritis/osteoarthritis/osteoporosis^**	582			
No		—	—	
Yes		-0.04	-0.11, 0.03	0.254
**Existing comorbidity: Hearing/visual impairment^**	582			
No		—	—	
Yes		-0.04	-0.15, 0.07	0.469
**Existing comorbidity: Other^**	582			
No		—	—	
Yes		-0.07	-0.13, -0.01	0.020
**From onset of stroke to inpatient rehabilitation (scaled days)^**	531	-0.04	-0.07, -0.02	<0.001
**Employment status prior to stroke**	582			
Retired		—	—	
Employed		0.16	0.10, 0.22	<0.001
Not employed		0.05	0.00, 0.11	0.073
**Experienced complications during rehabilitation**	582			
No		—	—	
Yes		-0.08	-0.14, -0.02	0.006
Unknown		-0.26	-0.46, -0.05	0.015
**FIM total score on rehabilitation admission (scaled)^**	582	0.12	0.08, 0.16	<0.001

[1]CI = Confidence Interval

**On stroke admission*

^On rehabilitation admission

Table SXV. Univariate mixed effects linear regression for Functional Independence Measure efficiency				
**Characteristic**	**N**	**Beta**	**95% CI**[1]	**p-value**
**Age group (years)***	494			
Under 75		—	—	
75-84		-0.01	-0.17, 0.14	0.862
85 or more		-0.16	-0.34, 0.01	0.070
**Sex***	494			
Male		—	—	
Female		-0.04	-0.17, 0.10	0.608
**Type of stroke***	494			
Stroke of unspecified type		—	—	
Ischaemic stroke		-0.05	-0.32, 0.21	0.679
Haemorrhagic stroke		0.18	-0.14, 0.50	0.273
**Treated in a stroke unit or ICU or CCU***	494			
No		—	—	
Yes		0.10	-0.04, 0.24	0.169
**Number of comorbidities***	494	-0.04	-0.12, 0.04	0.362
**Country of birth***	494			
Australia		—	—	
Foreign countries		-0.07	-0.36, 0.21	0.613
Unknown		-0.01	-0.15, 0.13	0.889
**Social economic Status***	492			
Below median		—	—	
Median and above		-0.05	-0.19, 0.09	0.471
**Walk independently on admission***	494			
Yes		—	—	
No		0.23	-0.07, 0.53	0.131
Unknown		0.16	-0.14, 0.45	0.297
**NIHSS group***	494			
mild		—	—	
moderate		0.04	-0.28, 0.35	0.825
severe		-0.23	-0.75, 0.29	0.385
Unknown		-0.27	-0.51, -0.03	0.030
**Indigenous status***	493			
No		—	—	
Yes		-0.13	-0.82, 0.55	0.700
**Modified Monash Model remoteness***	492			
Metropolitan area/regional centre		—	—	
Rural area		0.04	-0.10, 0.18	0.573
**Admitted in daytime or night-time***	494			
Daytime		—	—	
Nighttime		0.01	-0.13, 0.15	0.863
**Admitted in weekday or non weekday***	494			
Business day		—	—	
Weekend or Holiday		0.11	-0.05, 0.27	0.192
**Inpatient rehabilitation delayed^**	494			
No		—	—	
Yes		-0.17	-0.32, -0.02	0.030
Unknown		-0.10	-0.31, 0.11	0.345
**Days delayed in starting inpatient rehabilitation^**	415	-0.04	-0.07, -0.02	0.001
**Existing comorbidity: Cardiac disease^**	494			
No		—	—	
Yes		-0.16	-0.33, 0.01	0.067
**Existing comorbidity: Respiratory disease^**	494			
No		—	—	
Yes		0.01	-0.26, 0.28	0.946
**Existing comorbidity: Drug and Alcohol abuse^**	494			
No		—	—	
Yes		-0.04	-0.47, 0.38	0.848
**Existing comorbidity: Mental health problem^**	494			
No		—	—	
Yes		0.09	-0.16, 0.35	0.470
**Previous history of stroke^**	494			
No		—	—	

Table SXV. Univariate mixed effects linear regression for Functional Independence Measure efficiency				
**Characteristic**	**N**	**Beta**	**95% CI[1]**	**p-value**
Yes		-0.21	-0.44, 0.02	0.068
**Existing comorbidity: Diabetes mellites^**	494			
No		—	—	
Yes		-0.06	-0.28, 0.16	0.590
**Existing comorbidity: Morbid obesity^**	494			
No		—	—	
Yes		-0.31	-0.79, 0.18	0.214
**Existing comorbidity: Chronic pain^**	494			
No		—	—	
Yes		-0.23	-0.64, 0.18	0.272
**Existing comorbidity: Cancer^**	494			
No		—	—	
Yes		-0.12	-0.48, 0.24	0.515
**Existing comorbidity: Dementia^**	494			
No		—	—	
Yes		-0.03	-0.38, 0.32	0.880
**Existing comorbidity: Renal failure^**	494			
No		—	—	
Yes		0.16	-0.35, 0.67	0.537
**Existing comorbidity: Arthritis/osteoarthritis/osteoporosis^**	494			
No		—	—	
Yes		-0.01	-0.22, 0.20	0.926
**Existing comorbidity: Hearing/visual impairment^**	494			
No		—	—	
Yes		-0.19	-0.51, 0.14	0.254
**Existing comorbidity: Other^**	494			
No		—	—	
Yes		-0.10	-0.29, 0.09	0.308
**From onset of stroke to inpatient rehabilitation (scaled days)^**	451	-0.11	-0.20, -0.02	0.016
**Employment status prior to stroke**	494			
Retired		—	—	
Employed		0.17	-0.02, 0.35	0.078
Not employed		-0.03	-0.20, 0.15	0.776
**Experienced complications during rehabilitation**	494			
No		—	—	
Yes		-0.32	-0.50, -0.14	<0.001
Unknown		-0.90	-1.5, -0.33	0.002
**FIM total score on rehabilitation admission (scaled)^**	494	-0.37	-0.52, -0.22	<0.001

[1]CI = Confidence Interval

**On stroke admission*

^*On rehabilitation admission*

Table SXVI. Univariate Negative binomial regression for length of stay in rehabilitation				
**Characteristic**	**N**	**IRR**[1]	**95% CI**[1]	**p-value**
**Age group (years)***	494			
Under 75		—	—	
75-84		0.94	0.83, 1.08	0.381
85 or more		0.85	0.73, 0.98	0.027
**Sex***	494			
Male		—	—	
Female		1.03	0.92, 1.16	0.585
**Type of stroke***	494			
Stroke of unspecified type		—	—	
Ischaemic stroke		1.47	1.18, 1.82	<0.001
Haemorrhagic stroke		1.55	1.18, 2.02	0.001
**Treated in a stroke unit or ICU or CCU***	494			
No		—	—	
Yes		1.04	0.93, 1.17	0.496
**Number of comorbidities***	494	1.14	1.06, 1.23	<0.001
**Country of birth***	494			
Australia		—	—	
Foreign countries		1.15	0.91, 1.46	0.248
Unknown		1.10	0.98, 1.24	0.110
**Social economic Status***	492			
Below median		—	—	
Median and above		1.04	0.93, 1.17	0.497
**Walk independently on admission***	494			
Yes		—	—	
No		1.27	0.99, 1.62	0.058
Unknown		1.42	1.10, 1.80	0.006
**NIHSS group***	494			
mild		—	—	
moderate		1.21	0.93, 1.58	0.155
severe		2.05	1.37, 3.16	<0.001
Unknown		1.47	1.20, 1.79	<0.001
**Indigenous status***	493			
No		—	—	
Yes		1.32	0.78, 2.43	0.339
**Modified Monash Model remoteness***	492			
metropolitan area/regional centre		—	—	
Rural area		0.94	0.83, 1.06	0.292
**Admitted in daytime or night-time***	494			
Daytime		—	—	
Nighttime		1.09	0.97, 1.22	0.159
**Admitted in weekday or non weekday***	494			
Business day		—	—	
Weekend/Holiday		1.13	0.99, 1.29	0.076
**Inpatient rehabilitation delayed^**	494			
No		—	—	
Yes		1.22	1.08, 1.38	0.002
Unknown		1.34	1.14, 1.60	<0.001
**Days delayed in starting inpatient rehabilitation^**	415	1.03	1.00, 1.05	0.019
**Existing comorbidity: Cardiac disease^**	494			
No		—	—	
Yes		1.02	0.89, 1.18	0.792
**Existing comorbidity: Respiratory disease^**	494			
No		—	—	
Yes		0.96	0.77, 1.20	0.691
**Existing comorbidity: Drug and Alcohol abuse^**	494			
No		—	—	
Yes		1.21	0.86, 1.75	0.292
**Existing comorbidity: Mental health problem^**	494			
No		—	—	
Yes		0.97	0.79, 1.21	0.792
**Previous history of stroke^**	494			
No		—	—	

Table SXVI. Univariate Negative binomial regression for length of stay in rehabilitation				
**Characteristic**	**N**	**IRR**[1]	**95% CI**[1]	**p-value**
Yes		1.20	1.00, 1.46	0.058
**Existing comorbidity: Diabetes mellites^**	494			
No		—	—	
Yes		1.14	0.95, 1.38	0.153
**Existing comorbidity: Morbid obesity^**	494			
No		—	—	
Yes		0.98	0.67, 1.51	0.942
**Existing comorbidity: Chronic pain^**	494			
No		—	—	
Yes		1.25	0.90, 1.79	0.199
**Existing comorbidity: Cancer^**	494			
No		—	—	
Yes		0.92	0.69, 1.27	0.609
**Existing comorbidity: Dementia^**	494			
No		—	—	
Yes		0.72	0.54, 0.97	0.025
**Existing comorbidity: Renal failure^**	494			
No		—	—	
Yes		1.27	0.85, 1.99	0.261
**Existing comorbidity: Arthritis/osteoarthritis/osteoporosis^**	494			
No		—	—	
Yes		1.10	0.93, 1.31	0.258
**Existing comorbidity: Hearing/visual impairment^**	494			
No		—	—	
Yes		0.76	0.58, 1.01	0.050
**Existing comorbidity: Other^**	494			
No		—	—	
Yes		0.91	0.78, 1.07	0.236
**From onset of stroke to inpatient rehabilitation (scaled days)^**	451	1.20	1.10, 1.31	<0.001
**Employment status prior to stroke**	494			
Employed		—	—	
Not employed		1.19	0.99, 1.44	0.066
Retired		1.14	0.98, 1.33	0.095
**Experienced complications during rehabilitation**	494			
No		—	—	
Yes		1.60	1.39, 1.84	<0.001
Unknown		0.72	0.46, 1.20	0.180
**FIM total score on rehabilitation admission (scaled)^**	494	0.61	0.59, 0.64	<0.001

[1]IRR = Incidence Rate Ratio, CI = Confidence Interval

**On stroke admission*

^*On rehabilitation admission*

Table SXVII. Multivariate mixed effects linear regression for relative functional gain (Days delayed in starting inpatient rehabilitation)			
**Characteristic**	**Beta**	**95% CI[1]**	**p-value**
**Age group (years)***			
Under 75	—	—	
75-84	-0.07	-0.13, -0.01	0.021
85 or more	-0.14	-0.21, -0.07	<0.001
**Sex***			
Male	—	—	
Female	-0.01	-0.06, 0.04	0.636
**Type of stroke***			
Stroke of unspecified type	—	—	
Ischaemic stroke	-0.04	-0.13, 0.05	0.366
Haemorrhagic stroke	0.02	-0.09, 0.13	0.703
**Treated in a stroke unit or ICU or CCU***			
No	—	—	
Yes	0.05	-0.01, 0.11	0.105
**Walk independently on admission***			
Yes	—	—	
No	0.12	0.02, 0.22	0.014
Unknown	0.16	0.05, 0.26	0.003
**NIHSS group***			
mild	—	—	
moderate	-0.04	-0.14, 0.07	0.479
severe	-0.06	-0.22, 0.09	0.438
Unknown	-0.08	-0.17, 0.01	0.096
**Days delayed in starting inpatient rehabilitation^**	-0.01	-0.02, 0.00	0.043
**Number of comorbidities***	-0.01	-0.04, 0.01	0.304
**Previous history of stroke^**			
No	—	—	
Yes	-0.01	-0.10, 0.08	0.811
**Existing comorbidity: Cardiac disease^**			
No	—	—	
Yes	0.01	-0.05, 0.08	0.739
**Existing comorbidity: Cancer^**			
No	—	—	
Yes	-0.03	-0.14, 0.08	0.559
**Existing comorbidity: Dementia^**			
No	—	—	
Yes	-0.12	-0.24, 0.00	0.056
**Existing comorbidity: Other^**			
No	—	—	
Yes	-0.09	-0.15, -0.02	0.009
**Experienced complications during rehabilitation**			
No	—	—	
Yes	-0.09	-0.15, -0.03	0.005
**From onset of stroke to inpatient rehabilitation (scaled days)^**	-0.03	-0.06, 0.00	0.033
**FIM total score on rehabilitation admission (scaled)^**	0.08	0.03, 0.13	0.004
**Employment status prior to stroke**			
Retired	—	—	
Employed	0.02	-0.05, 0.10	0.522
Not employed	0.05	-0.04, 0.14	0.284

[1]CI = Confidence Interval

**On stroke admission*

^*On rehabilitation admission*

**Table SXVIII. Multivariate mixed effects linear regression for Functional Independence Measure efficiency (Days delayed in starting inpatient rehabilitation)**			
**Characteristic**	**Beta**	**95% CI[1]**	**p-value**
**Age group (years)***			
Under 75	—	—	
75-84	0.01	-0.15, 0.17	0.913
85 or more	-0.18	-0.36, 0.01	0.062
**Sex***			
Male	—	—	
Female	-0.04	-0.17, 0.09	0.531
**Treated in a stroke unit or ICU or CCU***			
No	—	—	
Yes	0.06	-0.10, 0.22	0.463
**Type of stroke***			
Stroke of unspecified type	—	—	
Ischaemic stroke	-0.23	-0.47, 0.00	0.053
Haemorrhagic stroke	0.10	-0.19, 0.40	0.489
**Number of comorbidities***	-0.03	-0.10, 0.05	0.500
**Walk independently on admission***			
Yes	—	—	
No	0.16	-0.10, 0.42	0.227
Unknown	0.31	0.03, 0.60	0.032
**NIHSS group***			
mild	—	—	
moderate	0.00	-0.27, 0.28	0.978
severe	-0.19	-0.63, 0.25	0.385
Unknown	-0.35	-0.58, -0.12	0.003
**Days delayed in starting inpatient rehabilitation^**	-0.03	-0.06, -0.01	0.012
**Existing comorbidity: Cardiac disease^**			
No	—	—	
Yes	-0.02	-0.19, 0.15	0.822
**Previous history of stroke^**			
No	—	—	
Yes	-0.11	-0.34, 0.13	0.379
**Experienced complications during rehabilitation**			
No	—	—	
Yes	-0.36	-0.54, -0.18	<0.001
**From onset of stroke to inpatient rehabilitation (scaled days)^**	-0.08	-0.17, 0.00	0.049
**FIM total score on rehabilitation admission (scaled)^**	-0.43	-0.58, -0.28	<0.001
**Employment status prior to stroke**			
Retired	—	—	
Employed	0.15	-0.04, 0.35	0.116
Not employed	0.01	-0.23, 0.25	0.936

[1]CI = Confidence Interval

**On stroke admission*

^On rehabilitation admission

Table SXIX. Negative binomial regression for length of stay in rehabilitation (Days delayed in starting inpatient rehabilitation)			
**Characteristic**	**IRR[1]**	**95% CI[1]**	**p-value**
**Age group (years)***			
Under 75	—	—	
75-84	0.90	0.81, 1.01	0.076
85 or more	0.83	0.73, 0.94	0.004
**Sex***			
Male	—	—	
Female	1.00	0.91, 1.10	0.992
**Type of stroke***			
Stroke of unspecified type	—	—	
Ischaemic stroke	1.28	1.08, 1.52	0.004
Haemorrhagic stroke	1.17	0.95, 1.44	0.146
**Treated in a stroke unit or ICU or CCU***			
No	—	—	
Yes	1.05	0.94, 1.18	0.354
**NIHSS group***			
mild	—	—	
moderate	1.12	0.92, 1.35	0.258
severe	1.02	0.76, 1.38	0.897
Unknown	1.28	1.08, 1.50	0.003
**Walk independently on admission***			
Yes	—	—	
No	0.93	0.77, 1.11	0.416
Unknown	1.01	0.83, 1.23	0.938
**Number of comorbidities***	0.97	0.92, 1.03	0.337
**Admitted in weekday or non weekday***			
Business day	—	—	
Weekend/Holiday	0.98	0.88, 1.08	0.655
**Days delayed in starting inpatient rehabilitation^**	1.01	0.99, 1.02	0.548
**Previous history of stroke^**			
No	—	—	
Yes	1.08	0.92, 1.27	0.379
**Existing comorbidity: Dementia^**			
No	—	—	
Yes	0.61	0.49, 0.77	<0.001
**Existing comorbidity: Hearing/visual impairment^**			
No	—	—	
Yes	0.85	0.69, 1.05	0.127
**From onset of stroke to inpatient rehabilitation (scaled days)^**	0.99	0.93, 1.05	0.673
**Experienced complications during rehabilitation**			
No	—	—	
Yes	1.26	1.12, 1.42	<0.001
**Employment status prior to stroke**			
Employed	—	—	
Not employed	1.12	0.93, 1.36	0.217
Retired	1.13	0.99, 1.29	0.080
**FIM total score on rehabilitation admission (scaled)^**	0.62	0.59, 0.65	<0.001

[1]IRR = Incidence Rate Ratio, CI = Confidence Interval

**On stroke admission*

^*On rehabilitation admission*

Table SXX. Negative binomial regression for length of stay in rehabilitation (Stratified Days delayed in starting inpatient rehabilitation)			
**Characteristic**	**IRR**[1]	**95% CI**[1]	**p-value**
**Age group (years)***			
Under 75	—	—	
75-84	0.90	0.81, 1.01	0.063
85 or more	0.83	0.73, 0.95	0.005
**Sex***			
Male	—	—	
Female	1.01	0.92, 1.10	0.854
**Type of stroke***			
Stroke of unspecified type	—	—	
Ischaemic stroke	1.26	1.06, 1.49	0.008
Haemorrhagic stroke	1.15	0.93, 1.41	0.200
**Treated in a stroke unit or ICU or CCU***			
No	—	—	
Yes	1.04	0.93, 1.16	0.505
**NIHSS group***			
mild	—	—	
moderate	1.11	0.91, 1.34	0.296
severe	0.99	0.74, 1.33	0.949
Unknown	1.27	1.08, 1.49	0.004
**Walk independently on admission***			
Yes	—	—	
No	0.93	0.78, 1.12	0.447
Unknown	1.00	0.82, 1.21	0.986
**Number of comorbidities***	0.98	0.93, 1.03	0.373
**Admitted in weekday or non weekday***			
Business day	—	—	
Weekend/Holiday	0.98	0.89, 1.09	0.739
**Days delayed in starting inpatient rehabilitation^**			
No delay	—	—	
Delayed for 1-2 days	1.15	1.04, 1.27	0.006
Delayed for 3 or more days	1.06	0.95, 1.19	0.309
**Previous history of stroke^**			
No	—	—	
Yes	1.05	0.90, 1.24	0.531
**Existing comorbidity: Dementia^**			
No	—	—	
Yes	0.62	0.50, 0.77	<0.001
**Existing comorbidity: Hearing/visual impairment^**			
No	—	—	
Yes	0.85	0.69, 1.05	0.123
**From onset of stroke to inpatient rehabilitation (scaled days)^**	1.00	0.94, 1.05	0.890
**Experienced complications during rehabilitation**			
No	—	—	
Yes	1.26	1.13, 1.42	<0.001
**Employment status prior to stroke**			
Employed	—	—	
Not employed	1.12	0.93, 1.35	0.217
Retired	1.12	0.98, 1.27	0.106
**FIM total score on rehabilitation admission (scaled)^**	0.62	0.59, 0.65	<0.001

[1]IRR = Incidence Rate Ratio, CI = Confidence Interval

**On stroke admission*
