# Supplementary material for: DELAYED INPATIENT REHABILITATION AND FUNCTIONAL OUTCOMES FOR ACUTE STROKE: A RETROSPECTIVE COHORT STUDY IN AN AUSTRALIAN REGIONAL HOSPITAL
Source: J Rehabil Med. 2025 Aug 5;57:42506. doi: 10.2340/jrm.v57.42506 (PMC12340994; doi:10.2340/jrm.v57.42506)
Supplement: DELAYED INPATIENT REHABILITATION AND FUNCTIONAL OUTCOMES FOR ACUTE STROKE: A RETROSPECTIVE COHORT STUDY IN AN AUSTRALIAN REGIONAL HOSPITAL [file JRM-57-42506-s3.pdf]

Supplementary material has been published as submitted. It has not been copyedited, or typeset by Journal of Rehabilitation Medicine

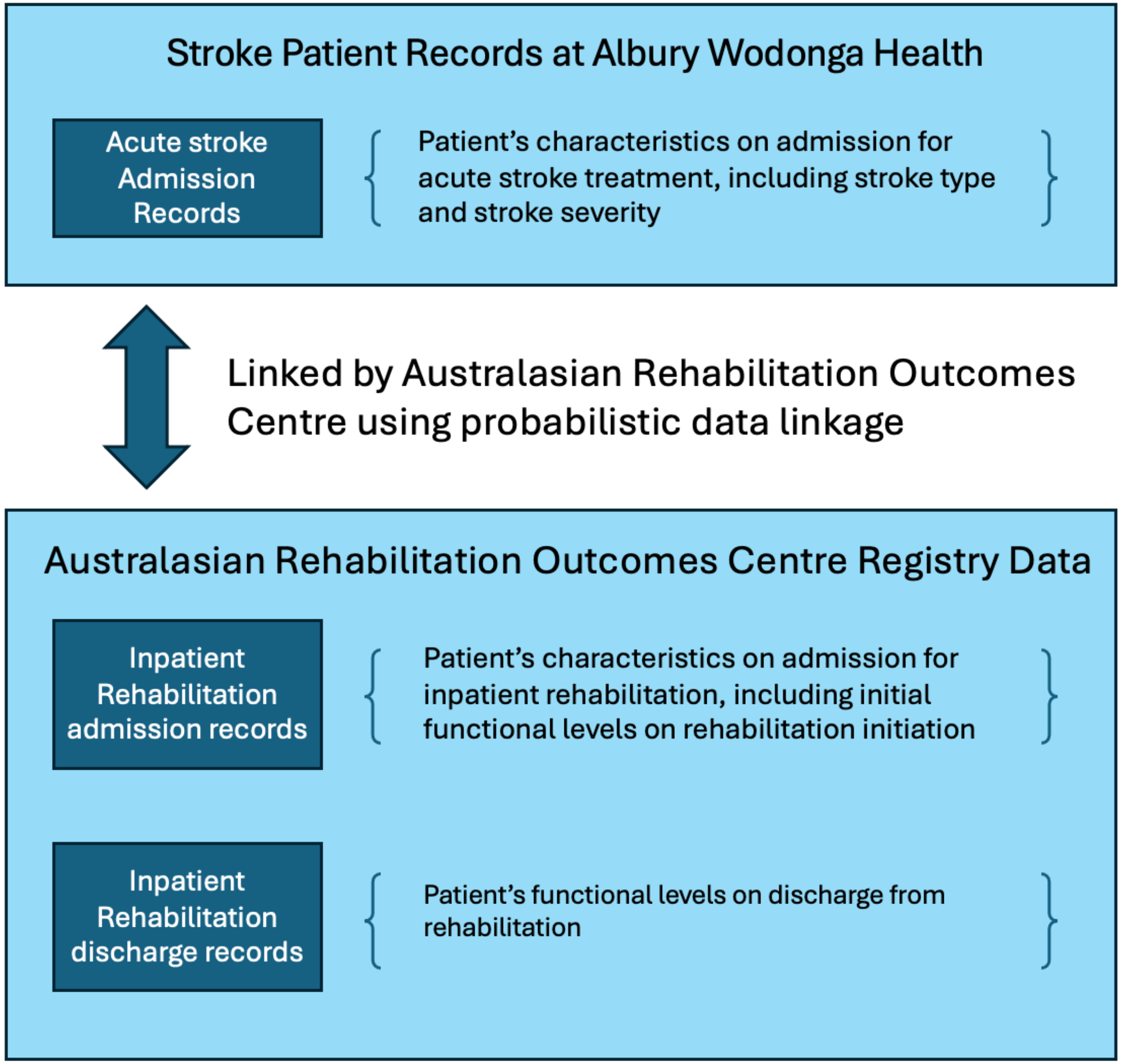

Figure S1. Linkage of Stroke Patient Records at AWH with AROC Registry Data

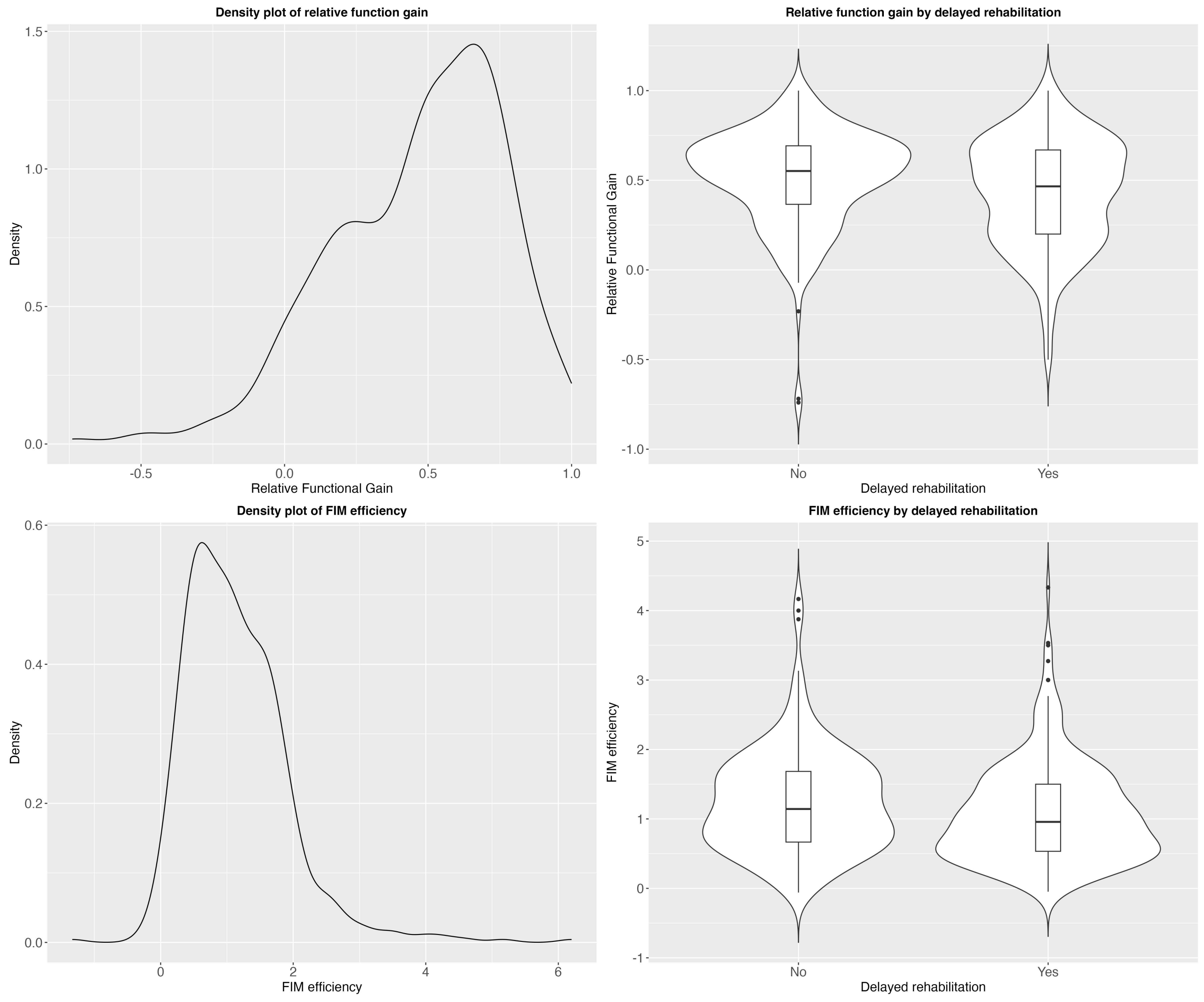

Figure S2. The distributions of the RFG and FIM efficiency

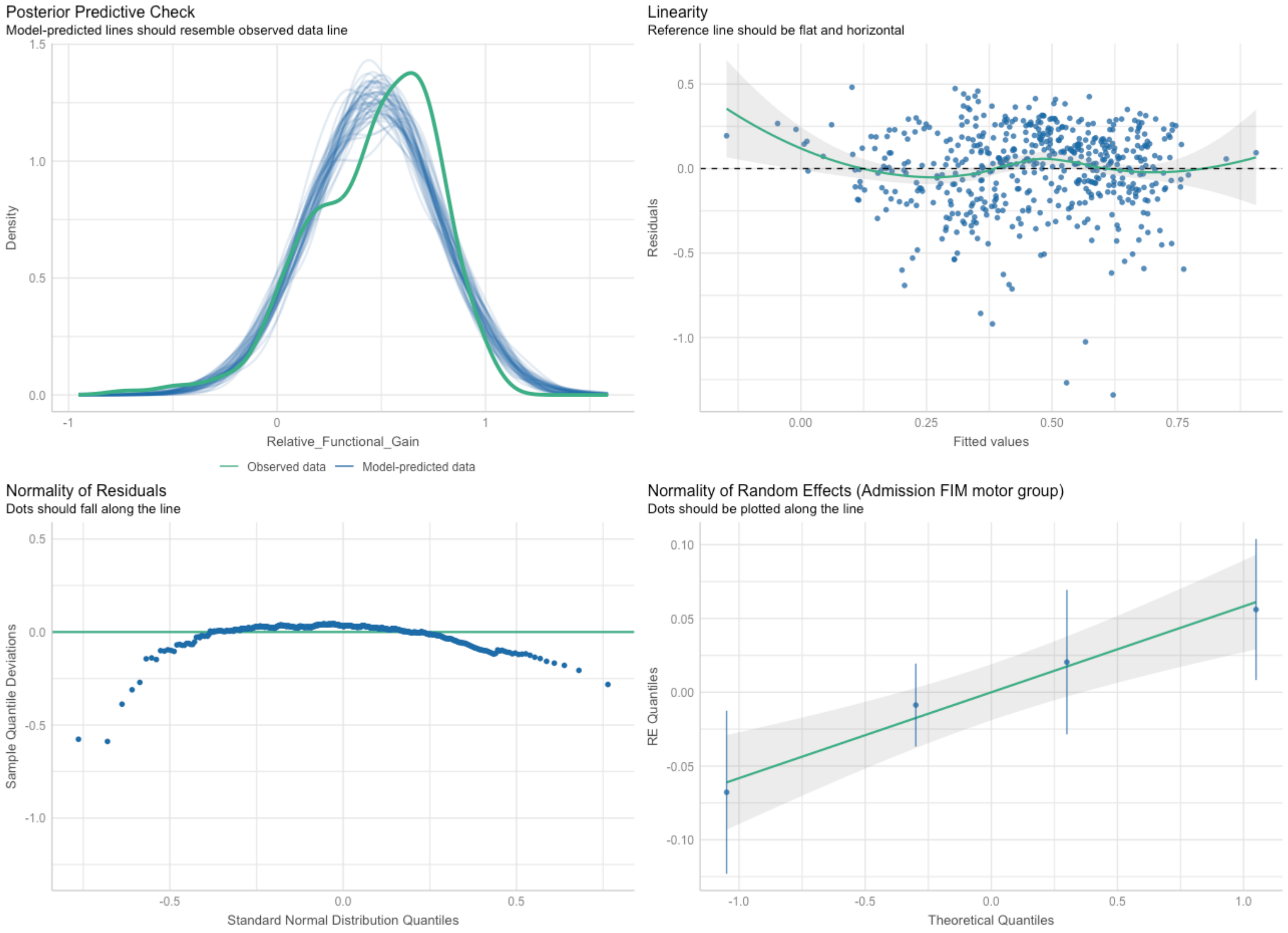

Figure S3. Diagnostic plots for Multivariate mixed effects linear regression for RFG (Inpatient rehabilitation delayed)

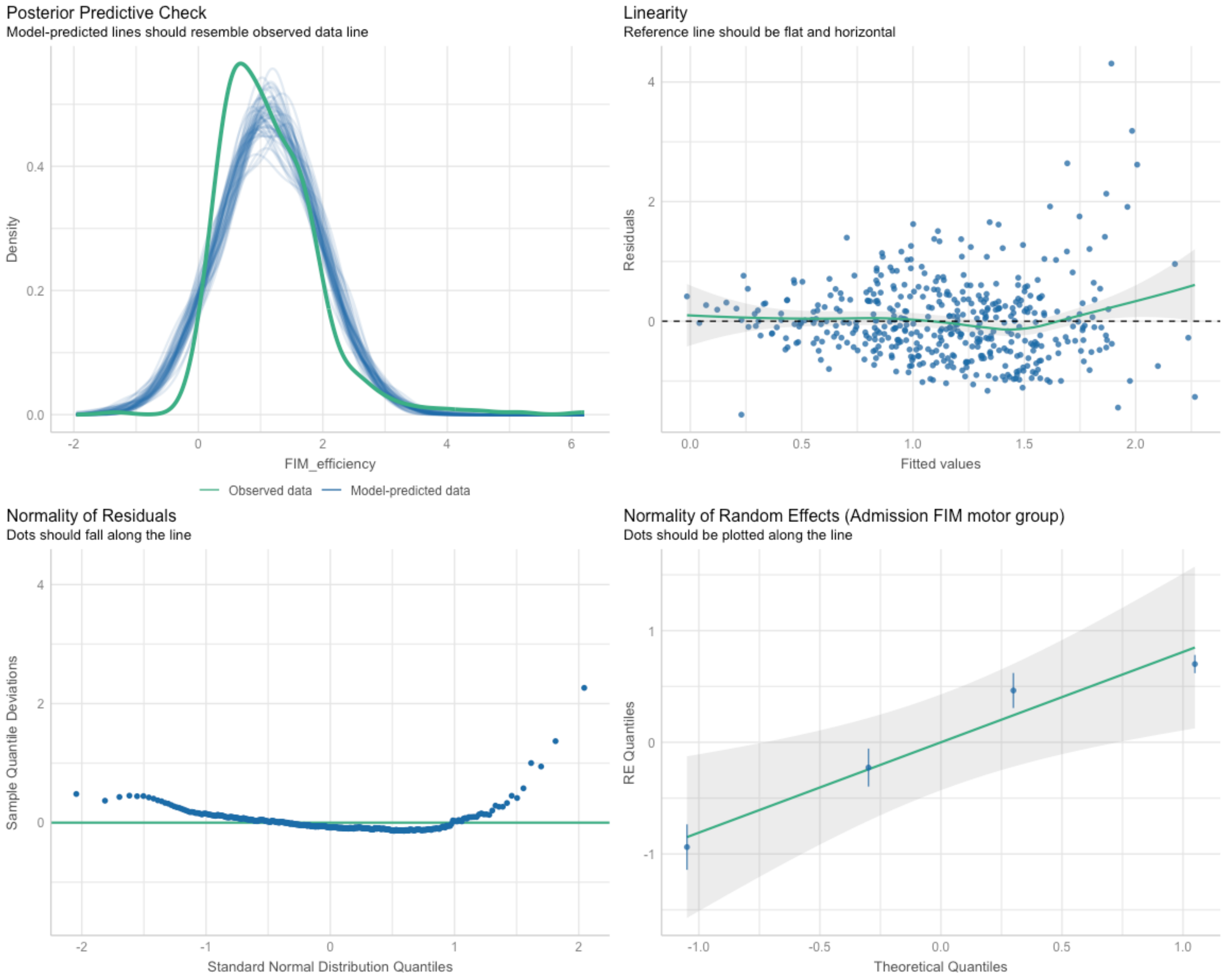

Figure S4. Diagnostic plots for Multivariate mixed effects linear regression for FIM efficiency (Inpatient rehabilitation delayed)

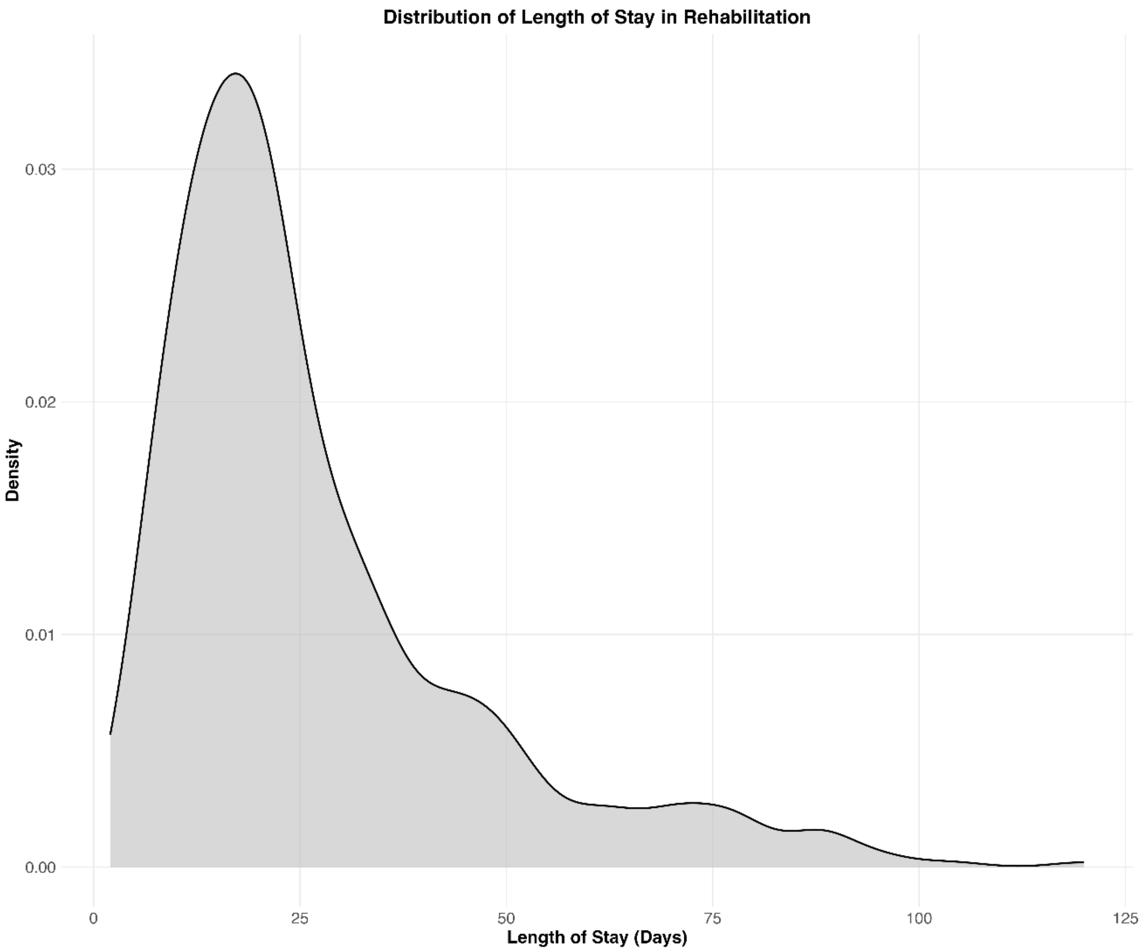

Figure S5. Density plot of length of stay in inpatient rehabilitation
